# Supplementary figures and images for: Arpin deficiency increases actomyosin contractility and vascular permeability (part 1 of 2)
Source: eLife. 2024 Sep 19;12:RP90692. doi: 10.7554/eLife.90692 (PMC11412691; doi:10.7554/eLife.90692)

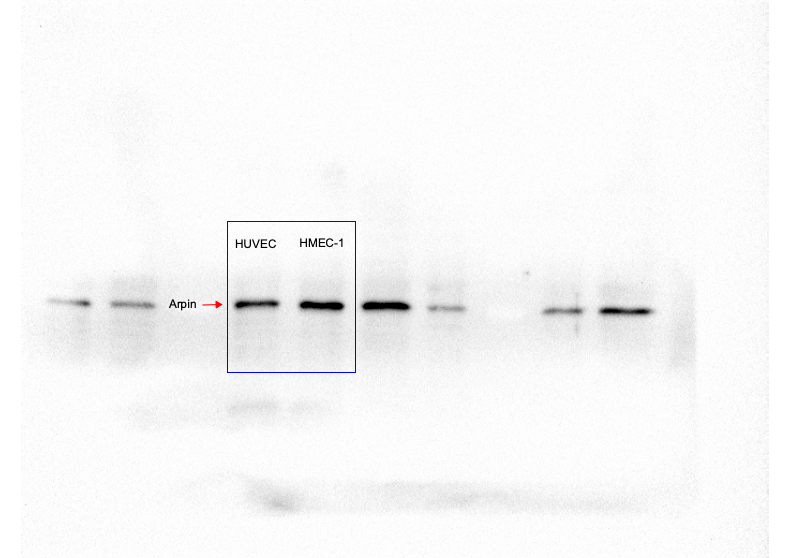

Supplement: Figure 1—source data 1. [file elife-90692-fig1-data1.zip › Figure 1 - Source data 1. Uncropped and labelled gels and membranes for Figure 1 /Fig 1B_Arpin_Labelled.tif]

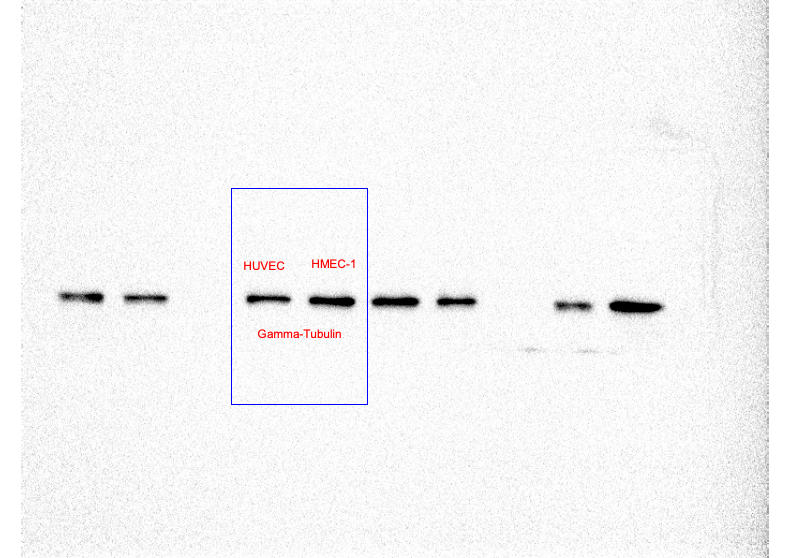

Supplement: Figure 1—source data 1. [file elife-90692-fig1-data1.zip › Figure 1 - Source data 1. Uncropped and labelled gels and membranes for Figure 1 /Fig 1B_Gamma Tubulin_Labelled.tif]

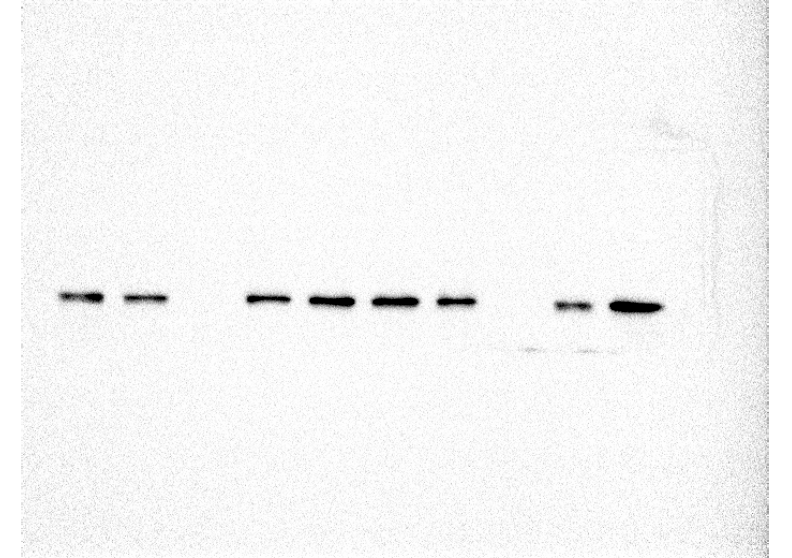

Supplement: Figure 1—source data 2. [file elife-90692-fig1-data2.zip › Figure 1 - Source data 2. Raw unedited membranes and gels for Figure 1 /Fig 1B_Gamma Tubulin.tif]

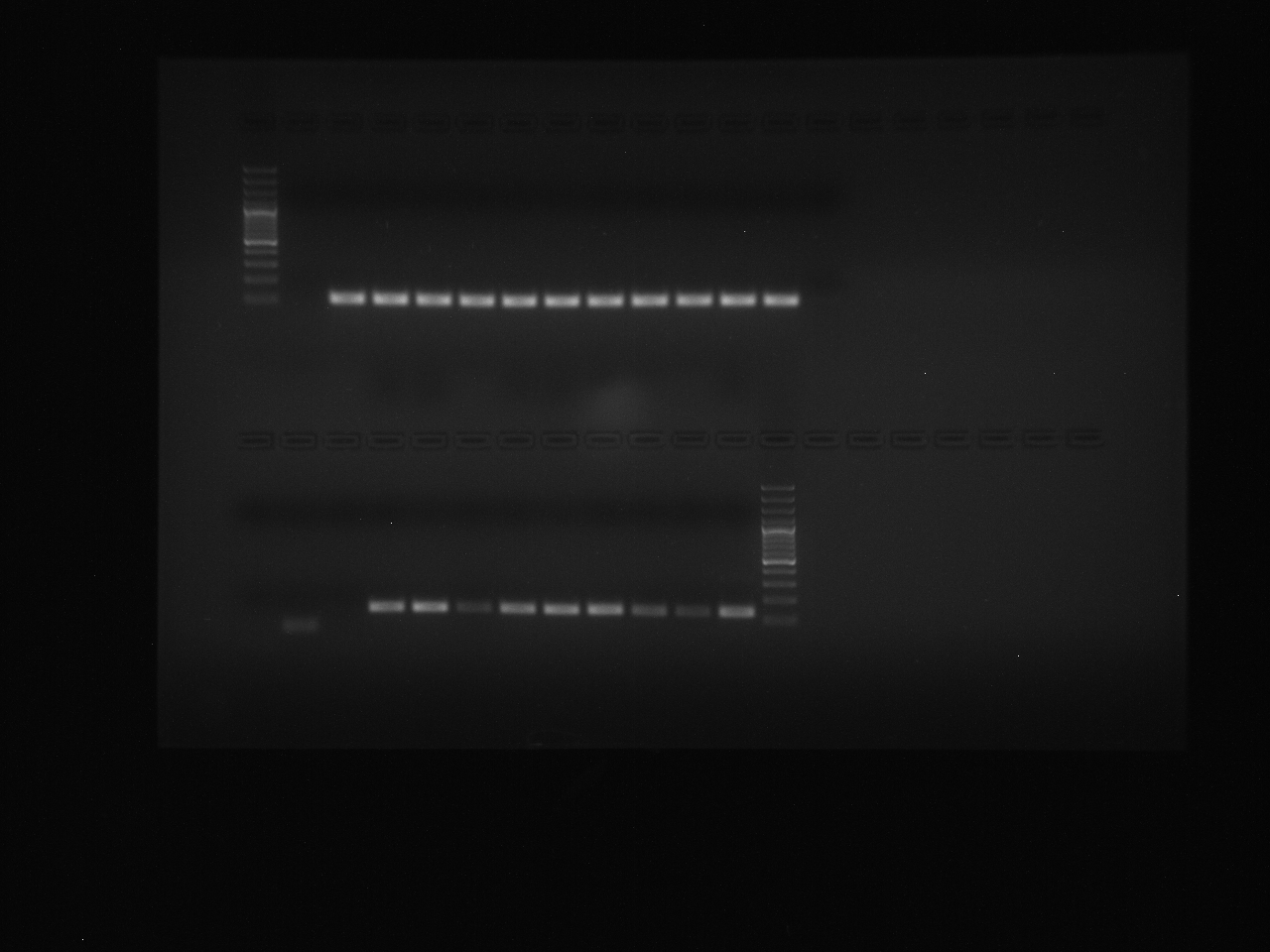

Supplement: Figure 1—source data 2. [file elife-90692-fig1-data2.zip › Figure 1 - Source data 2. Raw unedited membranes and gels for Figure 1 /Fig 1A_Arpin and Actb .tif]

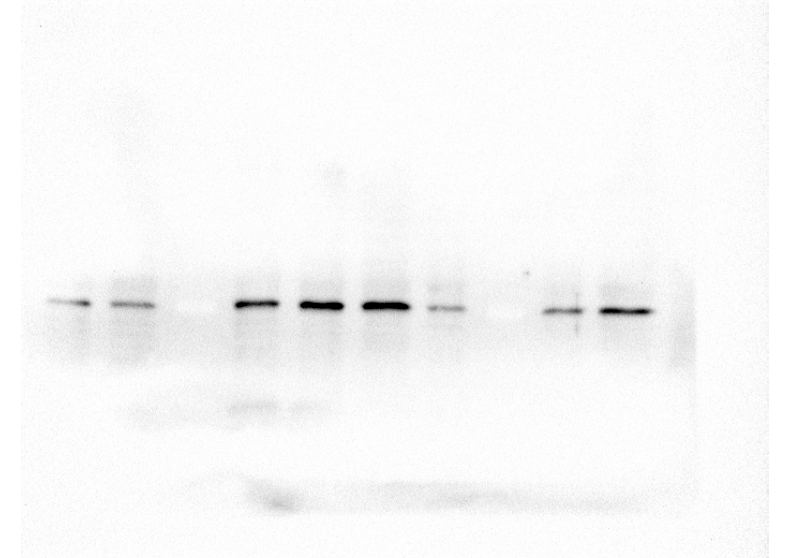

Supplement: Figure 1—source data 2. [file elife-90692-fig1-data2.zip › Figure 1 - Source data 2. Raw unedited membranes and gels for Figure 1 /Fig 1B_Arpin .tif]

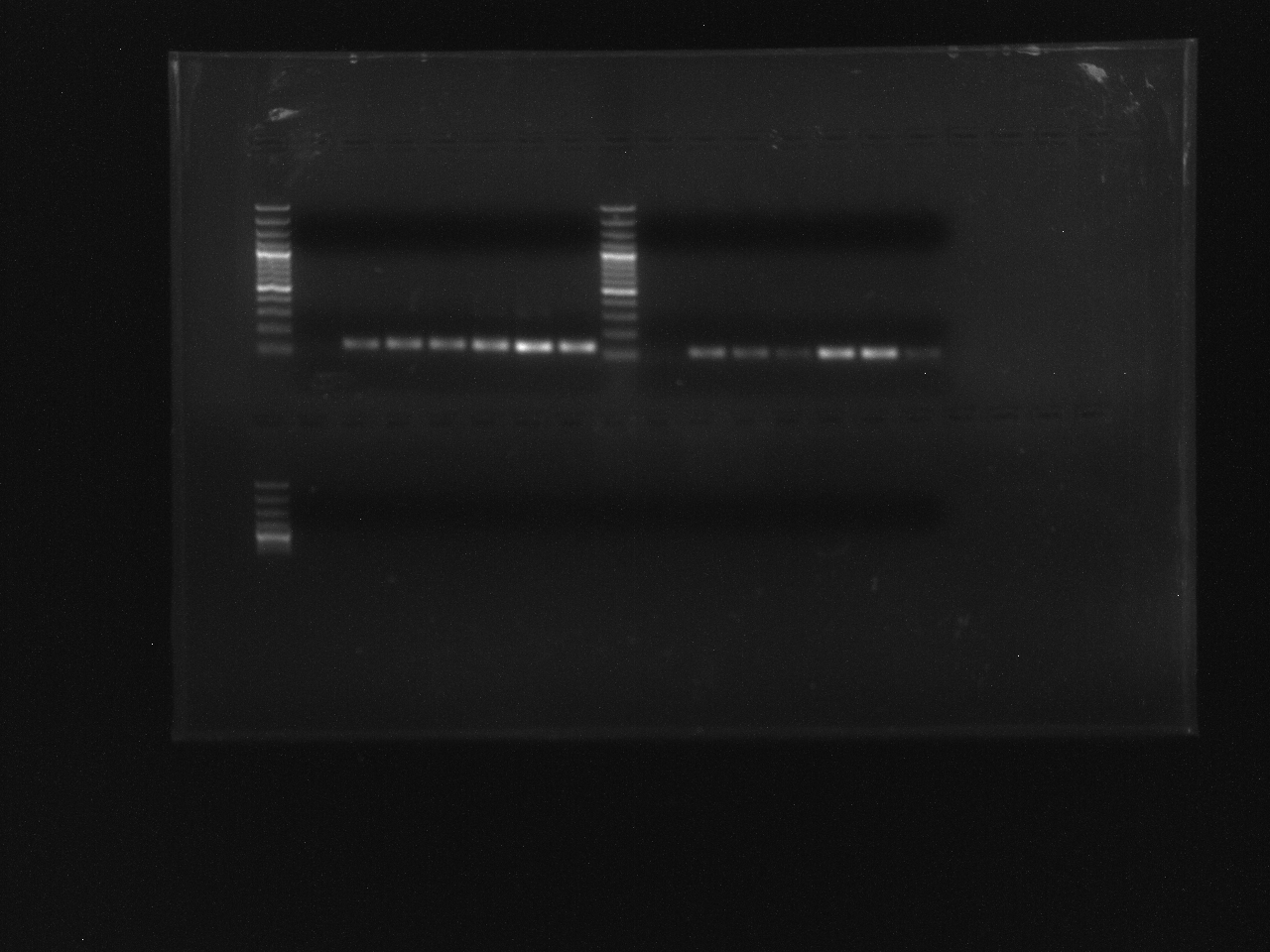

Supplement: Figure 1—source data 2. [file elife-90692-fig1-data2.zip › Figure 1 - Source data 2. Raw unedited membranes and gels for Figure 1 /Fig 1A_ARPIN and 7SL.tif]

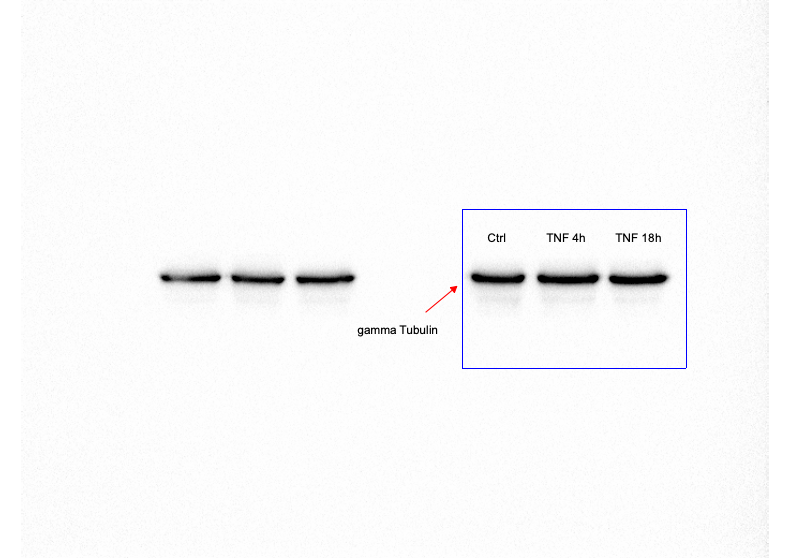

Supplement: Figure 2—source data 1. [file elife-90692-fig2-data1.zip › Figure 2 - Source data 1. Uncropped and labelled membranes for Figure 2/Fig 2B_Gamma Tubulin_Labelled.tif]

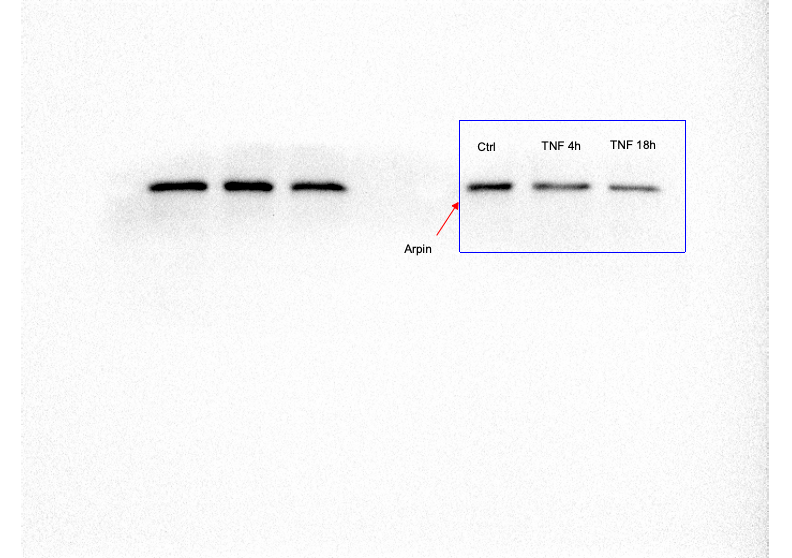

Supplement: Figure 2—source data 1. [file elife-90692-fig2-data1.zip › Figure 2 - Source data 1. Uncropped and labelled membranes for Figure 2/Fig 2B_Arpin_Labelled.tif]

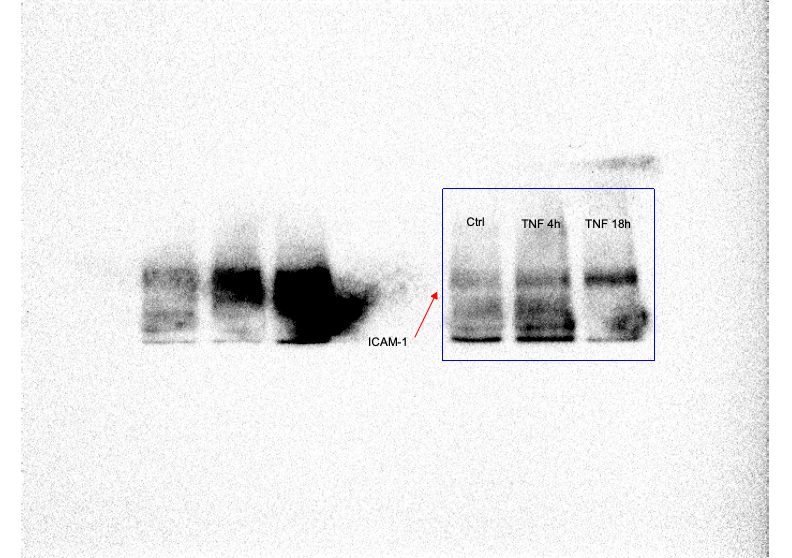

Supplement: Figure 2—source data 1. [file elife-90692-fig2-data1.zip › Figure 2 - Source data 1. Uncropped and labelled membranes for Figure 2/Fig 2B_ICAM-1_Labelled.tif]

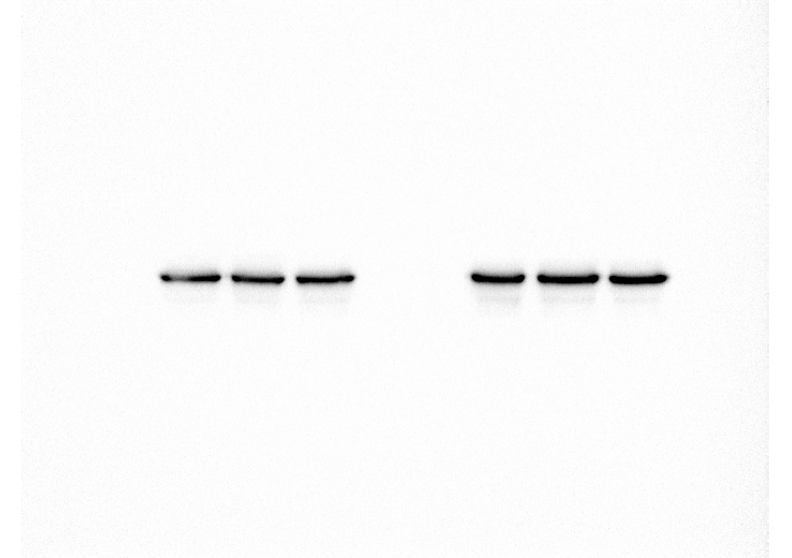

Supplement: Figure 2—source data 2. [file elife-90692-fig2-data2.zip › Figure 2 - Source data 2. Raw unedited membranes for Figure 2/Fig 2B_Gamma Tubulin .tif]

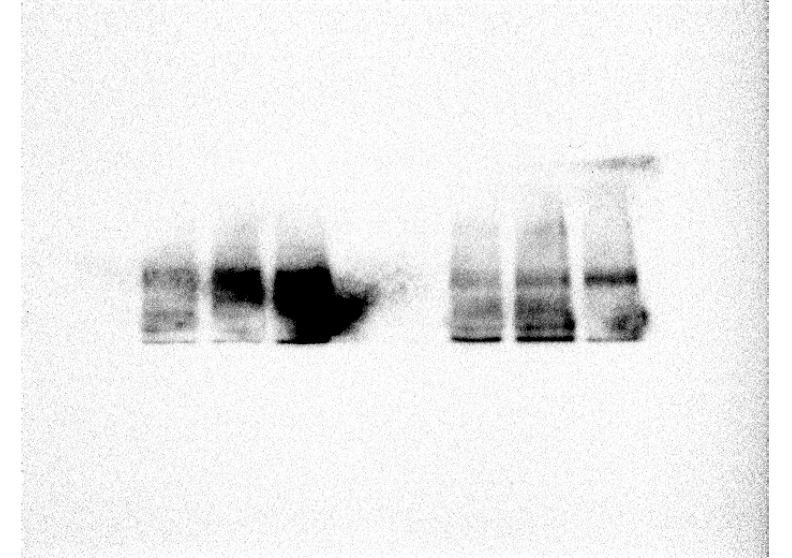

Supplement: Figure 2—source data 2. [file elife-90692-fig2-data2.zip › Figure 2 - Source data 2. Raw unedited membranes for Figure 2/Fig 2B_ICAM-1.tif]

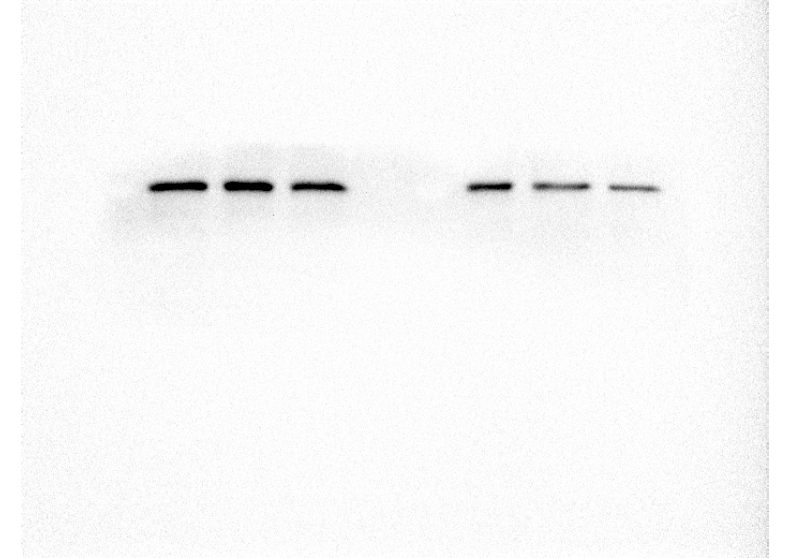

Supplement: Figure 2—source data 2. [file elife-90692-fig2-data2.zip › Figure 2 - Source data 2. Raw unedited membranes for Figure 2/Fig 2B_Arpin.tif]

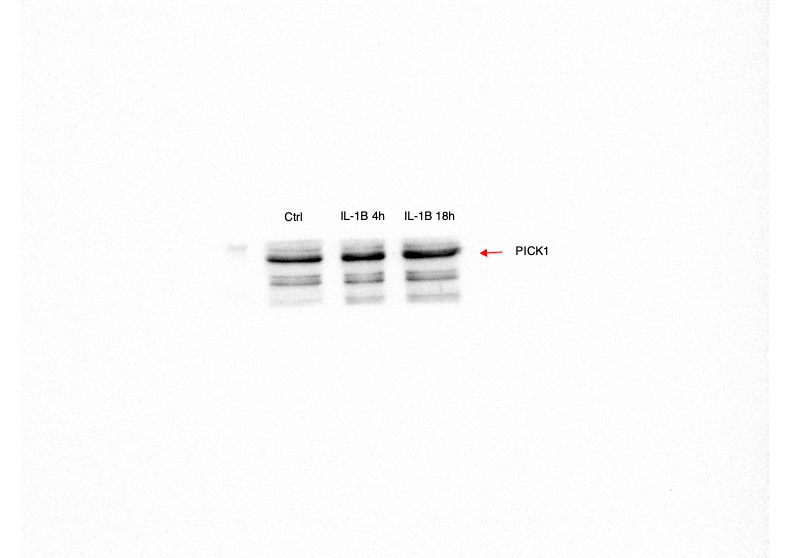

Supplement: Figure 2—figure supplement 1—source data 1. [file elife-90692-fig2-figsupp1-data1.zip › Figure 2 - Figure supplement 1 - Source data 1. Uncropped and labelled membranes for Figure 2 -Figure supplement 1/Fig 2 - FS1C - PICK1 - Labelled.tif]

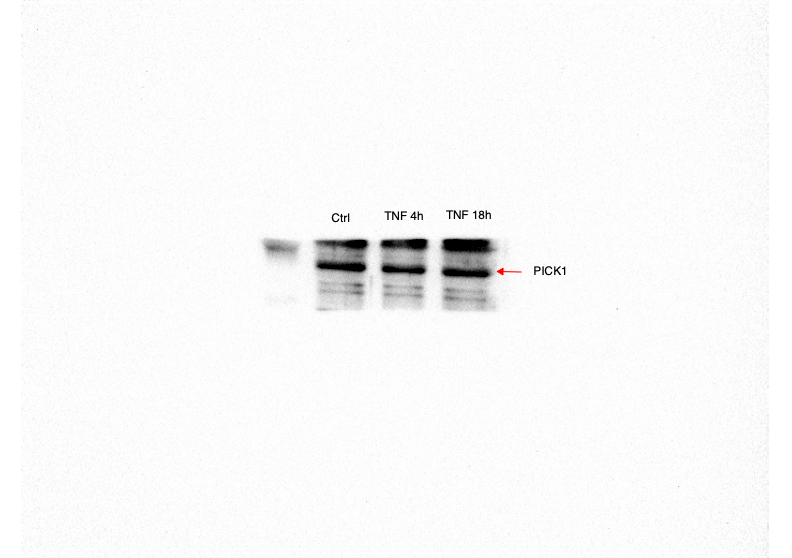

Supplement: Figure 2—figure supplement 1—source data 1. [file elife-90692-fig2-figsupp1-data1.zip › Figure 2 - Figure supplement 1 - Source data 1. Uncropped and labelled membranes for Figure 2 -Figure supplement 1/Fig 2 - FS1A - PICK1 - Labelled.tif]

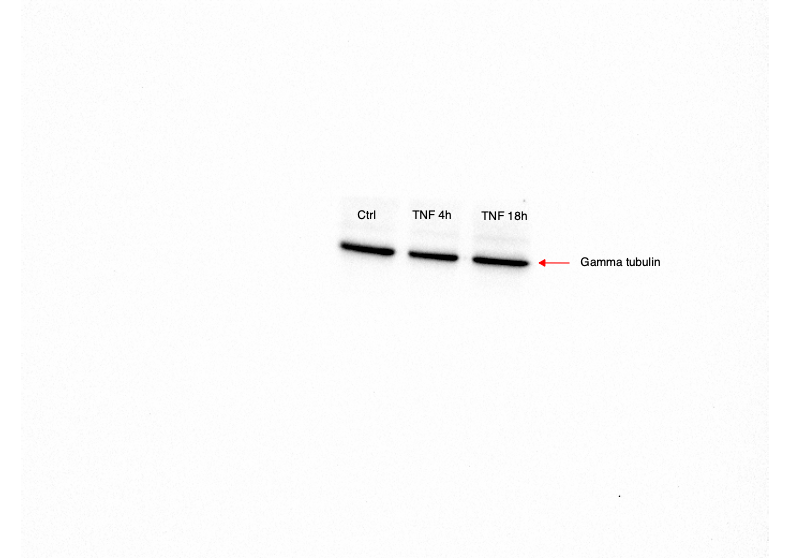

Supplement: Figure 2—figure supplement 1—source data 1. [file elife-90692-fig2-figsupp1-data1.zip › Figure 2 - Figure supplement 1 - Source data 1. Uncropped and labelled membranes for Figure 2 -Figure supplement 1/Fig 2 - FS1A - Gamma Tubulin - Labelled.tif]

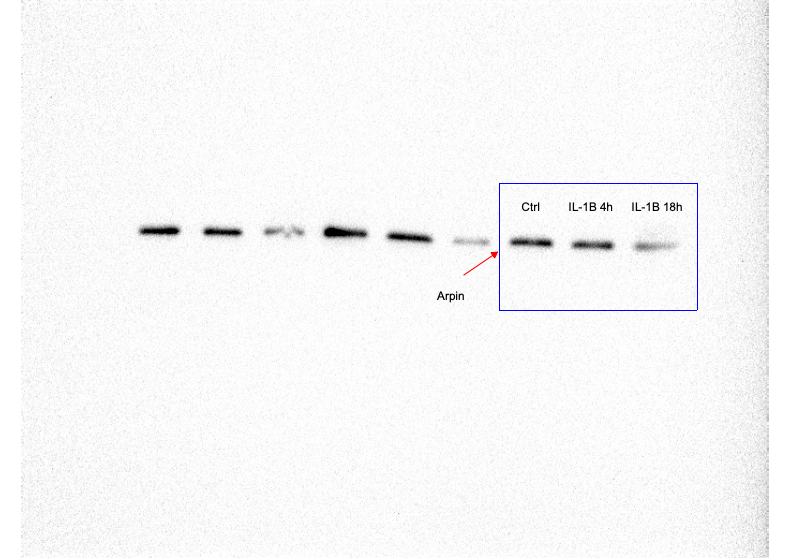

Supplement: Figure 2—figure supplement 1—source data 1. [file elife-90692-fig2-figsupp1-data1.zip › Figure 2 - Figure supplement 1 - Source data 1. Uncropped and labelled membranes for Figure 2 -Figure supplement 1/Fig 2 - FS1B - Arpin - Labelled.tif]

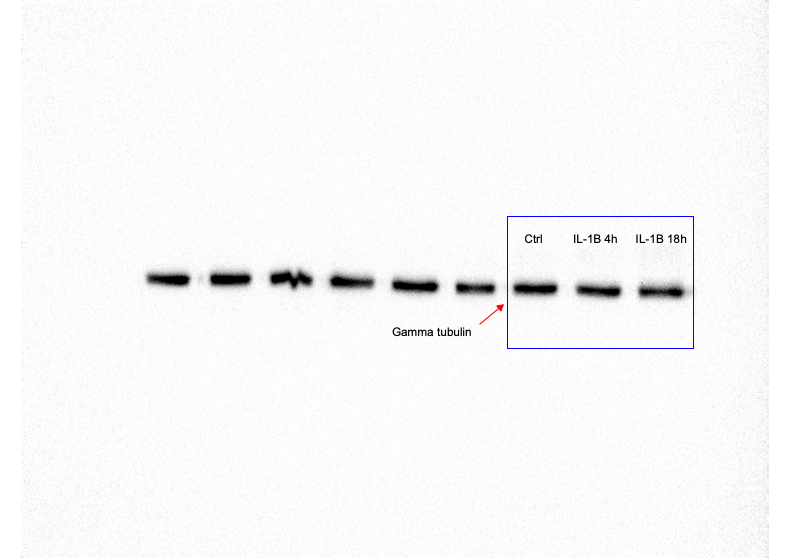

Supplement: Figure 2—figure supplement 1—source data 1. [file elife-90692-fig2-figsupp1-data1.zip › Figure 2 - Figure supplement 1 - Source data 1. Uncropped and labelled membranes for Figure 2 -Figure supplement 1/Fig 2 - FS1B - Gamma Tubulin - Labelled.tif]

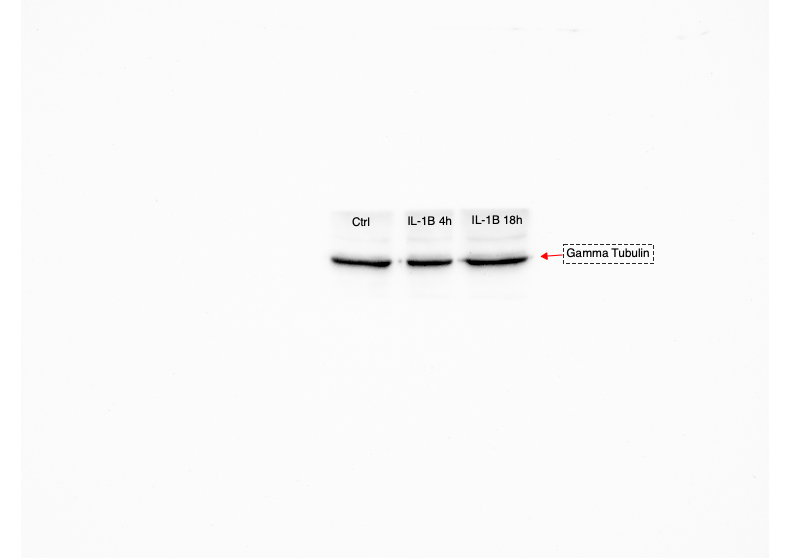

Supplement: Figure 2—figure supplement 1—source data 1. [file elife-90692-fig2-figsupp1-data1.zip › Figure 2 - Figure supplement 1 - Source data 1. Uncropped and labelled membranes for Figure 2 -Figure supplement 1/Fig 2 - FS1C - Gamma Tubulin - Labelled.tif]

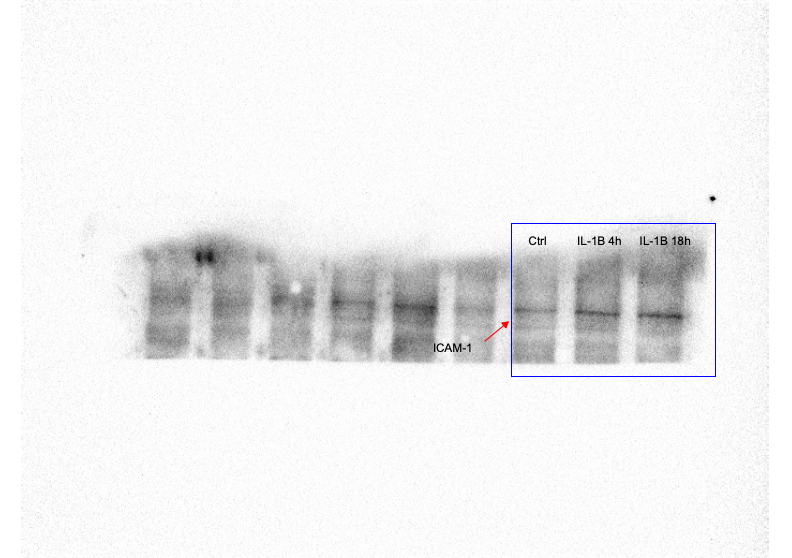

Supplement: Figure 2—figure supplement 1—source data 1. [file elife-90692-fig2-figsupp1-data1.zip › Figure 2 - Figure supplement 1 - Source data 1. Uncropped and labelled membranes for Figure 2 -Figure supplement 1/Fig 2 - FS1B - ICAM-1 - Labelled.tif]

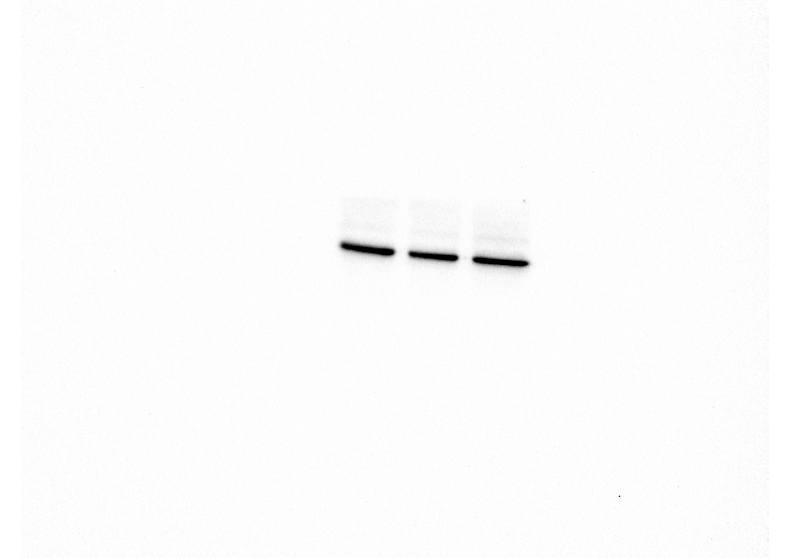

Supplement: Figure 2—figure supplement 1—source data 2. [file elife-90692-fig2-figsupp1-data2.zip › Figure 2 - Figure supplement 1 - Source Figure 2. Raw unedited membranes for Figure 2 - Figure supplement 1/Fig 2 - FS1A - Gamma Tubulin.tif]

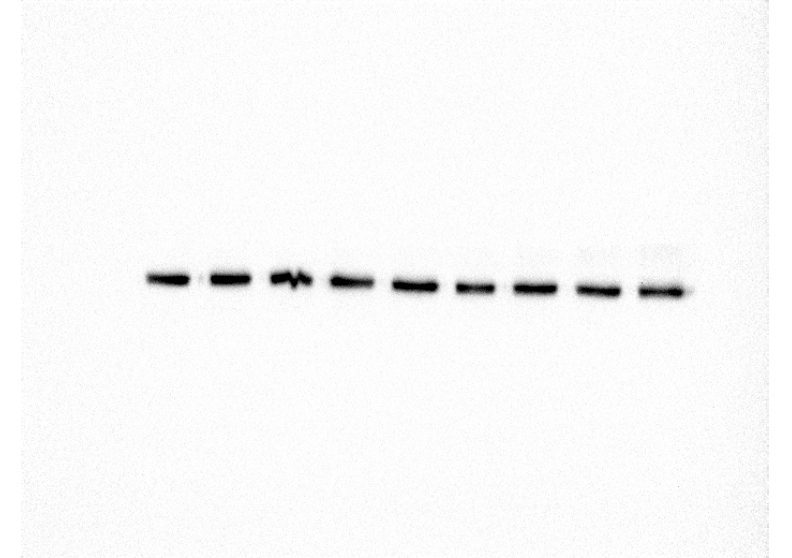

Supplement: Figure 2—figure supplement 1—source data 2. [file elife-90692-fig2-figsupp1-data2.zip › Figure 2 - Figure supplement 1 - Source Figure 2. Raw unedited membranes for Figure 2 - Figure supplement 1/Fig 2 - FS1B - Gamma Tubulin.tif]

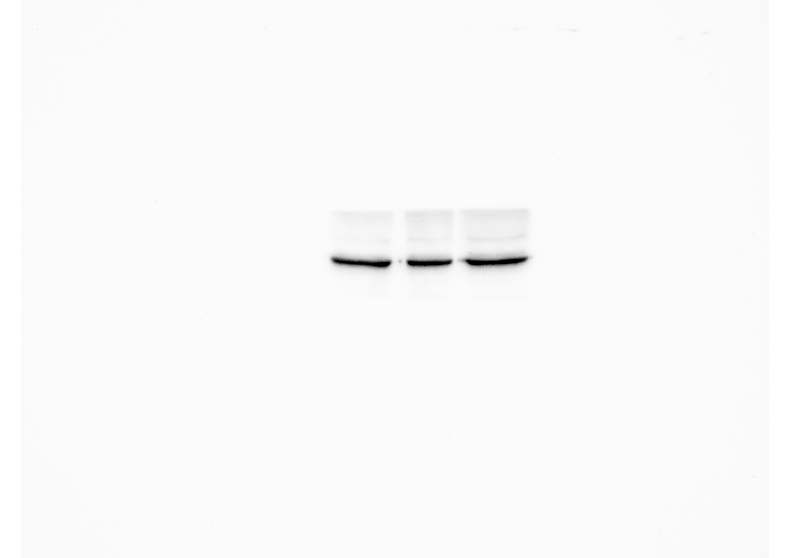

Supplement: Figure 2—figure supplement 1—source data 2. [file elife-90692-fig2-figsupp1-data2.zip › Figure 2 - Figure supplement 1 - Source Figure 2. Raw unedited membranes for Figure 2 - Figure supplement 1/Fig 2 - FS1C - Gamma Tubulin.tif]

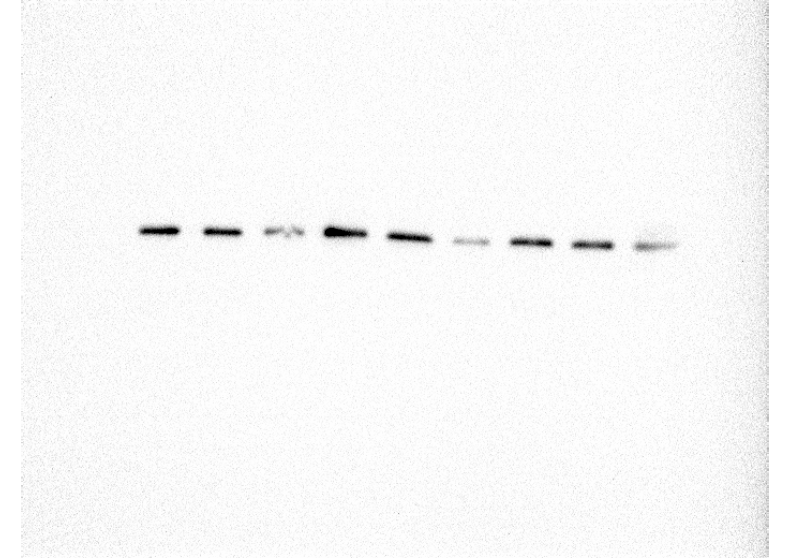

Supplement: Figure 2—figure supplement 1—source data 2. [file elife-90692-fig2-figsupp1-data2.zip › Figure 2 - Figure supplement 1 - Source Figure 2. Raw unedited membranes for Figure 2 - Figure supplement 1/Fig 2 - FS1B - Arpin.tif]

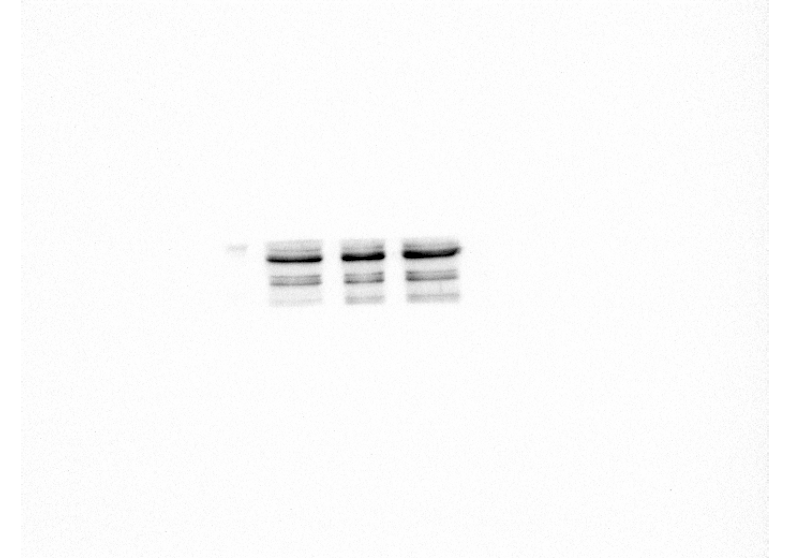

Supplement: Figure 2—figure supplement 1—source data 2. [file elife-90692-fig2-figsupp1-data2.zip › Figure 2 - Figure supplement 1 - Source Figure 2. Raw unedited membranes for Figure 2 - Figure supplement 1/Fig 2 - FS1C - PICK1.tif]

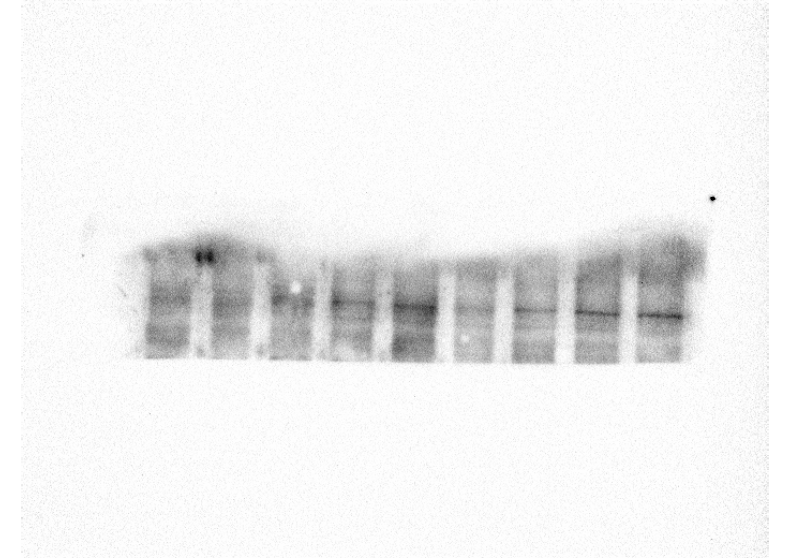

Supplement: Figure 2—figure supplement 1—source data 2. [file elife-90692-fig2-figsupp1-data2.zip › Figure 2 - Figure supplement 1 - Source Figure 2. Raw unedited membranes for Figure 2 - Figure supplement 1/Fig 2 - FS1B - ICAM-1.tif]

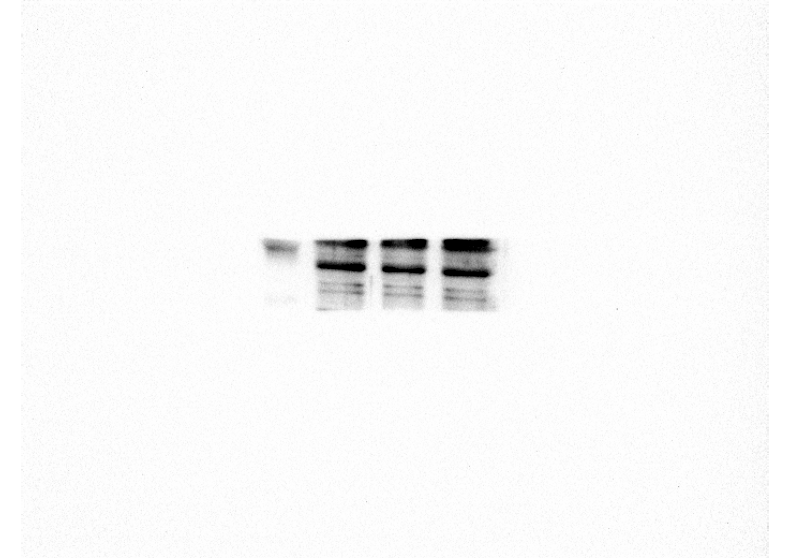

Supplement: Figure 2—figure supplement 1—source data 2. [file elife-90692-fig2-figsupp1-data2.zip › Figure 2 - Figure supplement 1 - Source Figure 2. Raw unedited membranes for Figure 2 - Figure supplement 1/Fig 2 - FS1A - PICK1.tif]

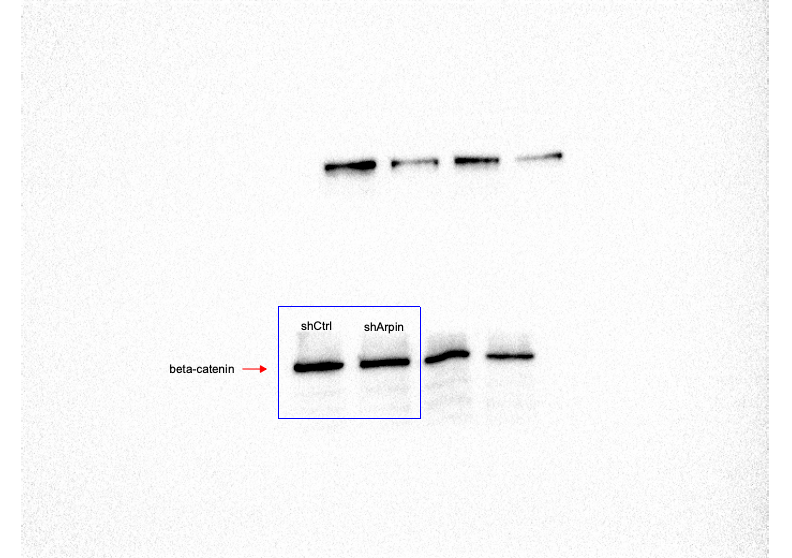

Supplement: Figure 3—source data 1. [file elife-90692-fig3-data1.zip › Figure 3 - Source data 1. Uncropped and labelled membranes for Figure 3/Fig 3C_B-catenin_Labelled.tif]

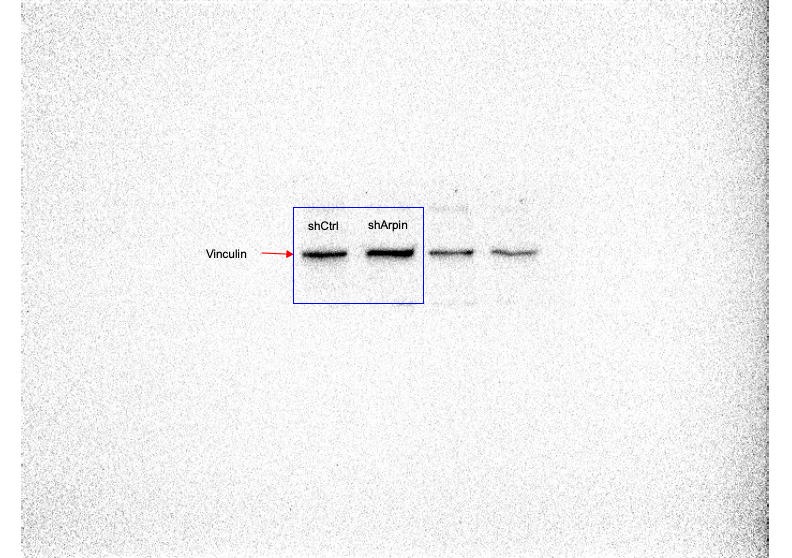

Supplement: Figure 3—source data 1. [file elife-90692-fig3-data1.zip › Figure 3 - Source data 1. Uncropped and labelled membranes for Figure 3/Fig 3C_Vinculin_Labelled.tif]

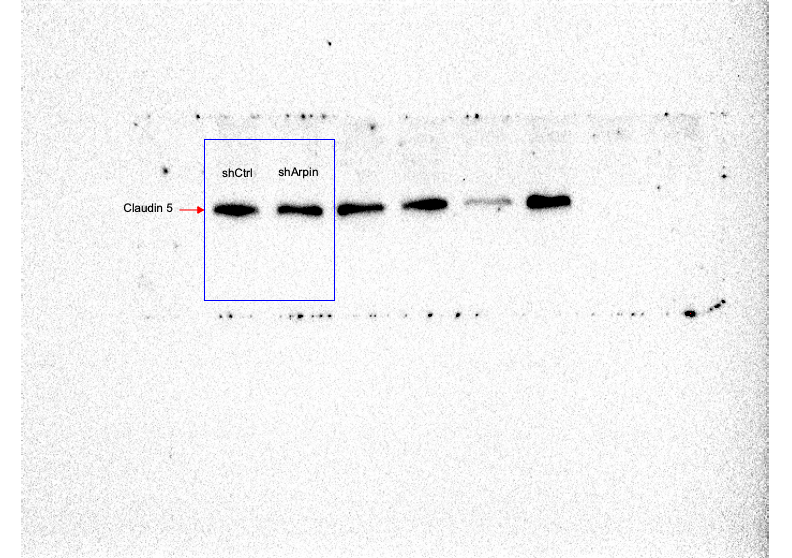

Supplement: Figure 3—source data 1. [file elife-90692-fig3-data1.zip › Figure 3 - Source data 1. Uncropped and labelled membranes for Figure 3/Fig 3D_Claudin-5_Labelled.tif]

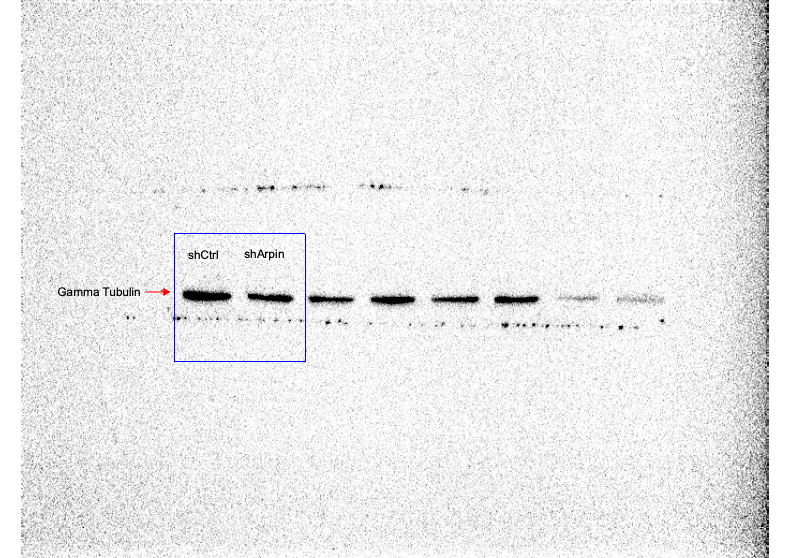

Supplement: Figure 3—source data 1. [file elife-90692-fig3-data1.zip › Figure 3 - Source data 1. Uncropped and labelled membranes for Figure 3/Fig 3D_Gamma Tubulin_Labelled.tif]

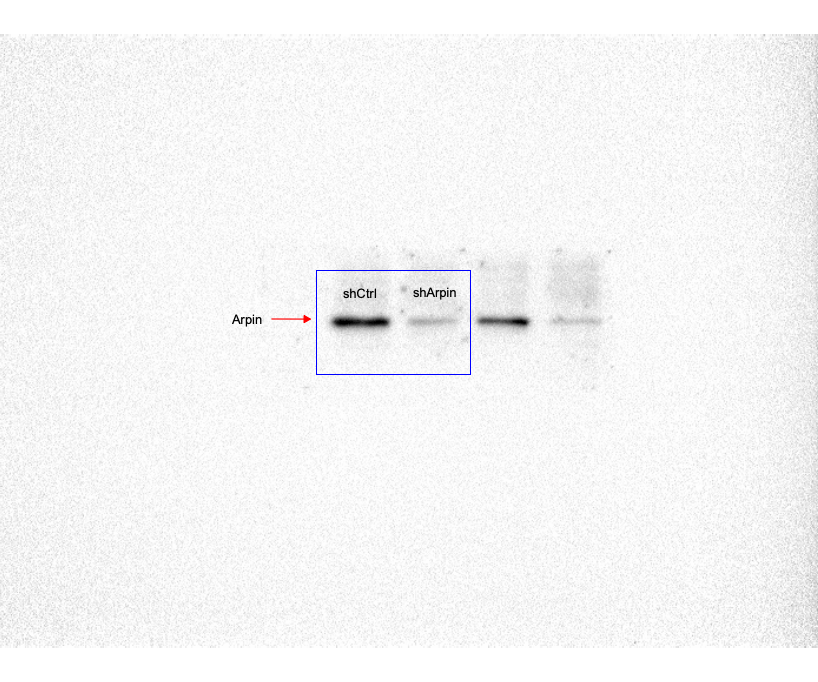

Supplement: Figure 3—source data 1. [file elife-90692-fig3-data1.zip › Figure 3 - Source data 1. Uncropped and labelled membranes for Figure 3/Fig 3C_Arpin_Labelled.tif]

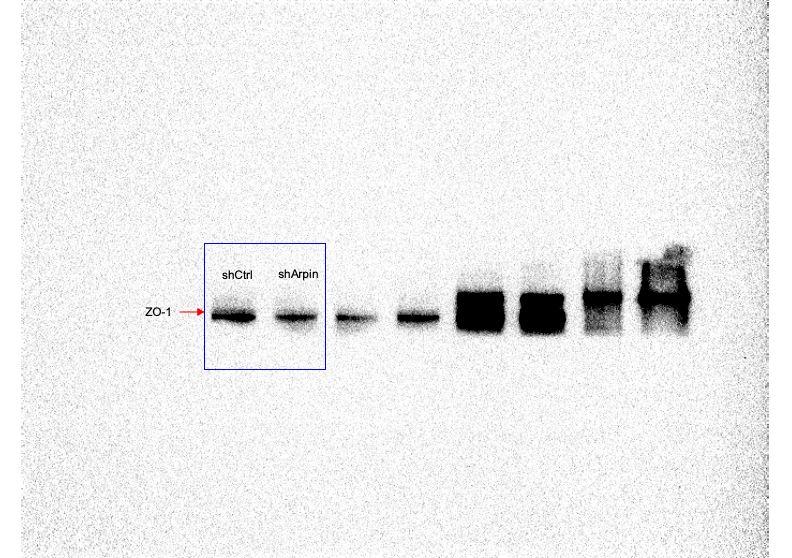

Supplement: Figure 3—source data 1. [file elife-90692-fig3-data1.zip › Figure 3 - Source data 1. Uncropped and labelled membranes for Figure 3/Fig 3D_ZO-1_Labelled.tif]

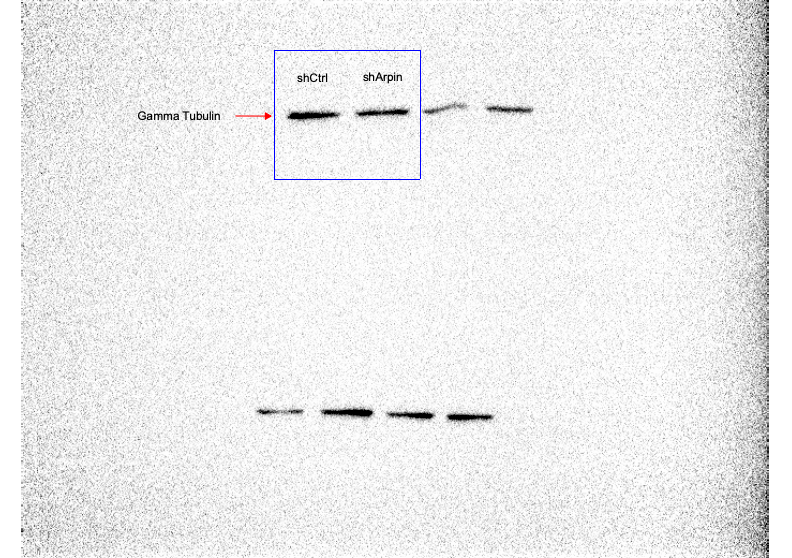

Supplement: Figure 3—source data 1. [file elife-90692-fig3-data1.zip › Figure 3 - Source data 1. Uncropped and labelled membranes for Figure 3/Fig 3C_Gamma Tubulin_Labelled.tif]

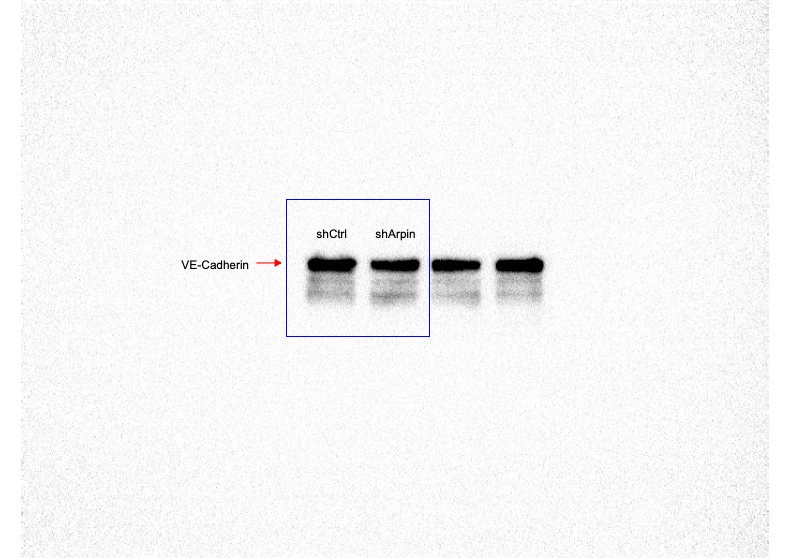

Supplement: Figure 3—source data 1. [file elife-90692-fig3-data1.zip › Figure 3 - Source data 1. Uncropped and labelled membranes for Figure 3/Fig 3C_VE-Cadherin_Labelled.tif]

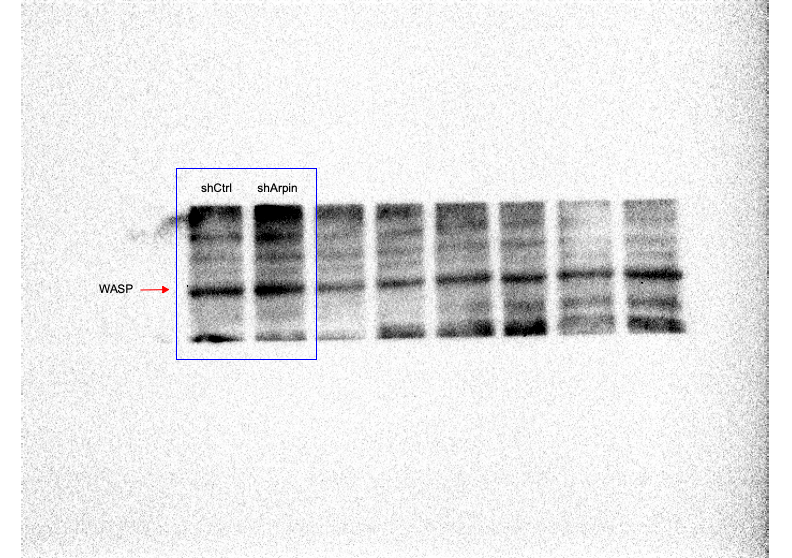

Supplement: Figure 3—source data 1. [file elife-90692-fig3-data1.zip › Figure 3 - Source data 1. Uncropped and labelled membranes for Figure 3/Fig 3A_WASP_Labelled.tif]

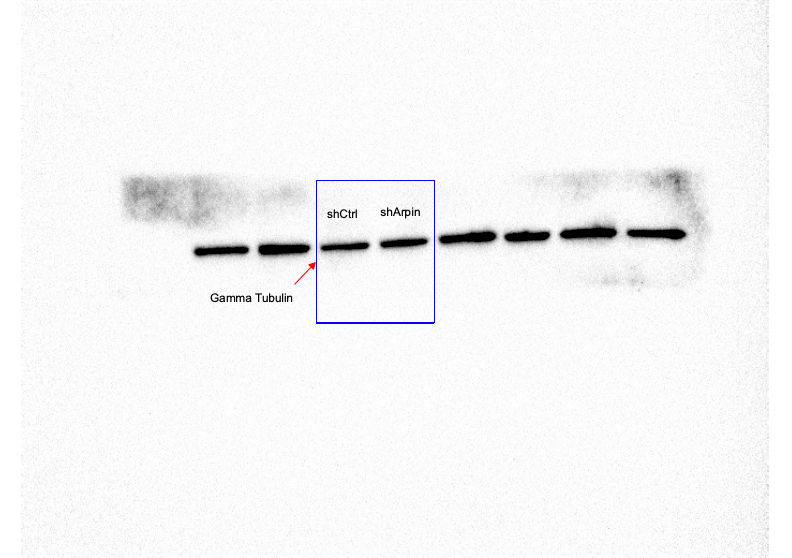

Supplement: Figure 3—source data 1. [file elife-90692-fig3-data1.zip › Figure 3 - Source data 1. Uncropped and labelled membranes for Figure 3/Fig 3A_Gamma Tubulin_Labelled.tif]

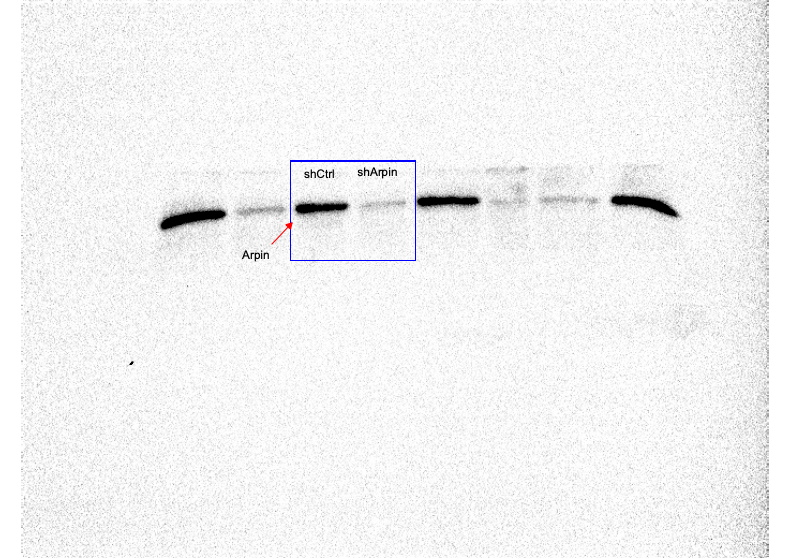

Supplement: Figure 3—source data 1. [file elife-90692-fig3-data1.zip › Figure 3 - Source data 1. Uncropped and labelled membranes for Figure 3/Fig 3A_Arpin_Labelled.tif]

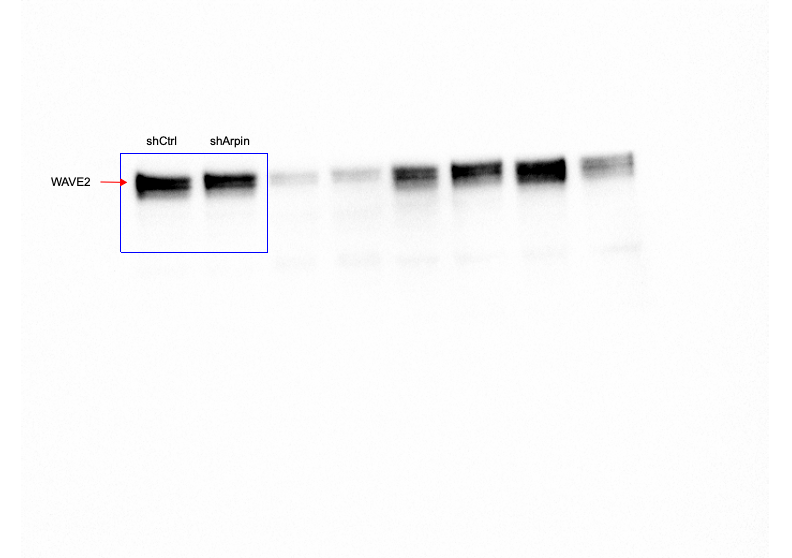

Supplement: Figure 3—source data 1. [file elife-90692-fig3-data1.zip › Figure 3 - Source data 1. Uncropped and labelled membranes for Figure 3/Fig 3A_WAVE2_Labelled.tif]

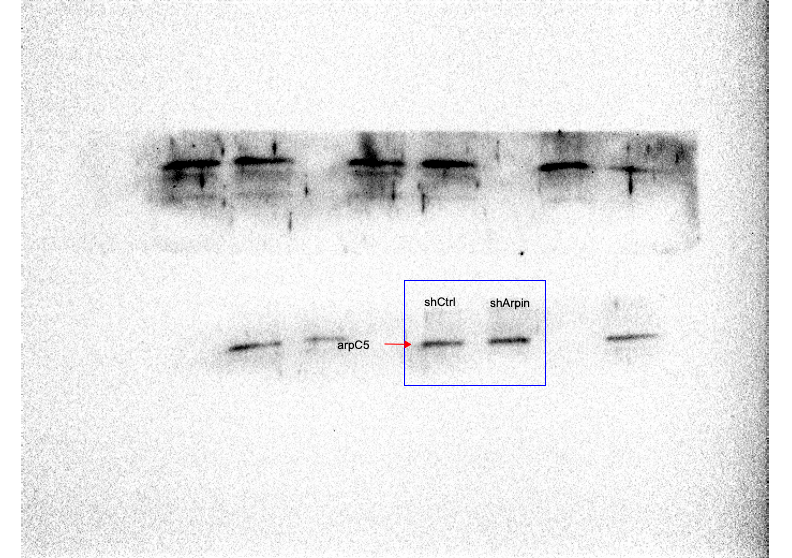

Supplement: Figure 3—source data 1. [file elife-90692-fig3-data1.zip › Figure 3 - Source data 1. Uncropped and labelled membranes for Figure 3/Fig 3A_arpC5_Labelled.tif]

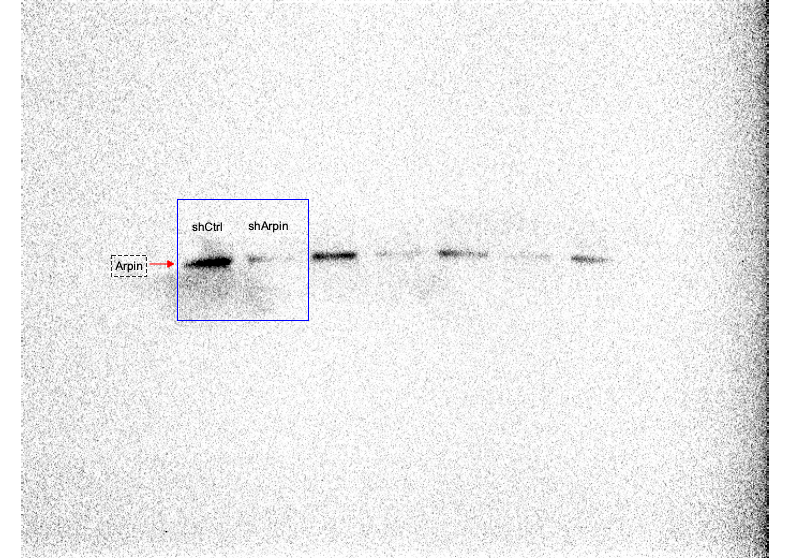

Supplement: Figure 3—source data 1. [file elife-90692-fig3-data1.zip › Figure 3 - Source data 1. Uncropped and labelled membranes for Figure 3/Fig 3D_Arpin_Labelled.tif]

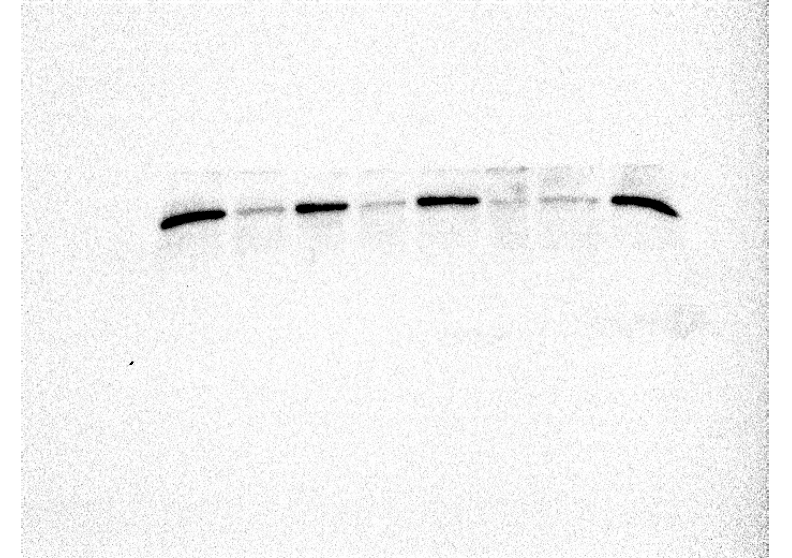

Supplement: Figure 3—source data 2. [file elife-90692-fig3-data2.zip › Figure 3 - Source data 2. Raw unedited membranes for Figure 3/Fig 3A_Arpin.tif]

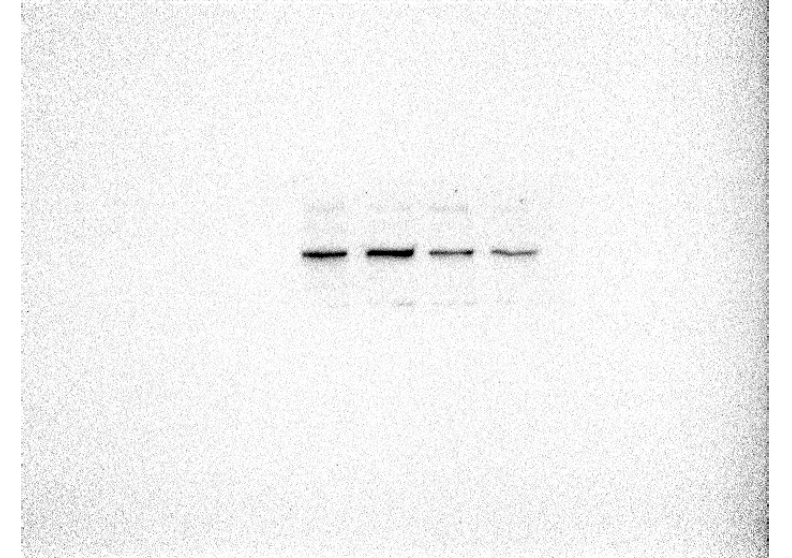

Supplement: Figure 3—source data 2. [file elife-90692-fig3-data2.zip › Figure 3 - Source data 2. Raw unedited membranes for Figure 3/Fig 3C_Vinculin.tif]

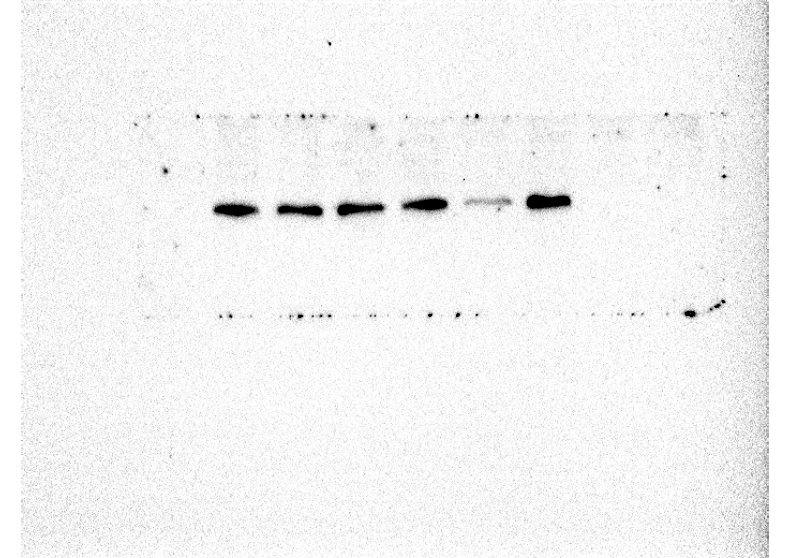

Supplement: Figure 3—source data 2. [file elife-90692-fig3-data2.zip › Figure 3 - Source data 2. Raw unedited membranes for Figure 3/Fig 3D_Claudin-5.tif]

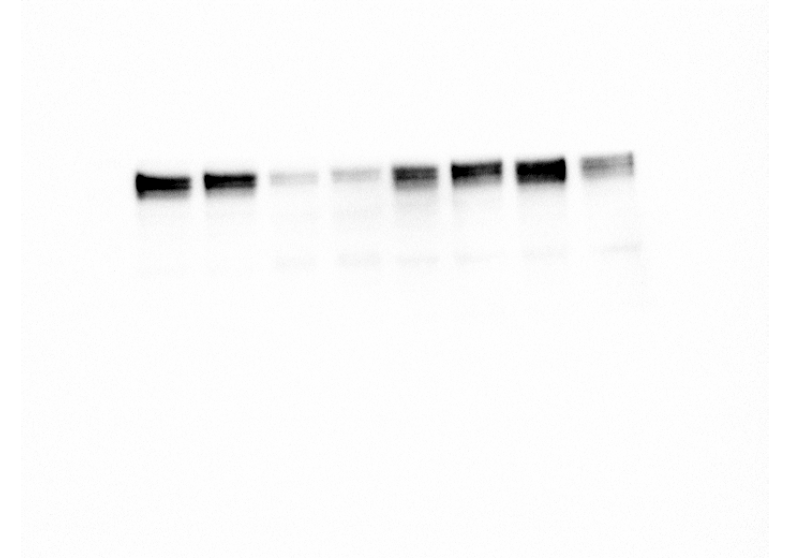

Supplement: Figure 3—source data 2. [file elife-90692-fig3-data2.zip › Figure 3 - Source data 2. Raw unedited membranes for Figure 3/Fig 3A_WAVE2.tif]

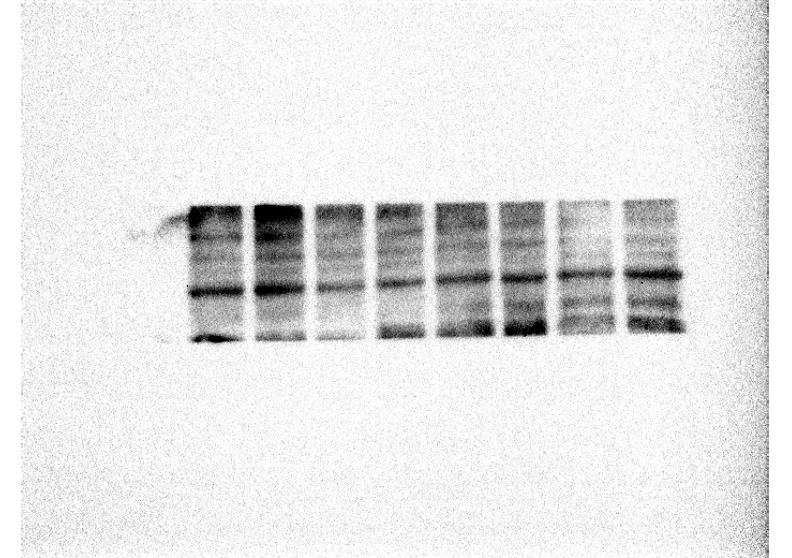

Supplement: Figure 3—source data 2. [file elife-90692-fig3-data2.zip › Figure 3 - Source data 2. Raw unedited membranes for Figure 3/Fig 3A_WASP.tif]

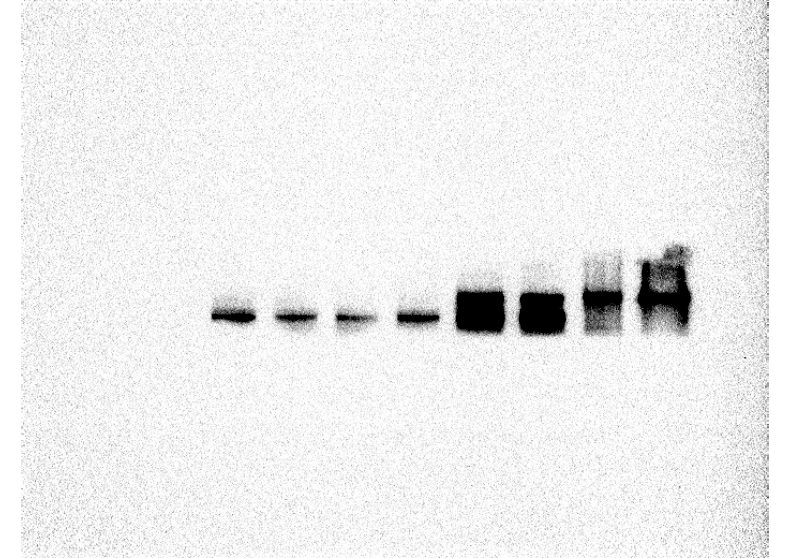

Supplement: Figure 3—source data 2. [file elife-90692-fig3-data2.zip › Figure 3 - Source data 2. Raw unedited membranes for Figure 3/Fig 3D_ZO-1.tif]

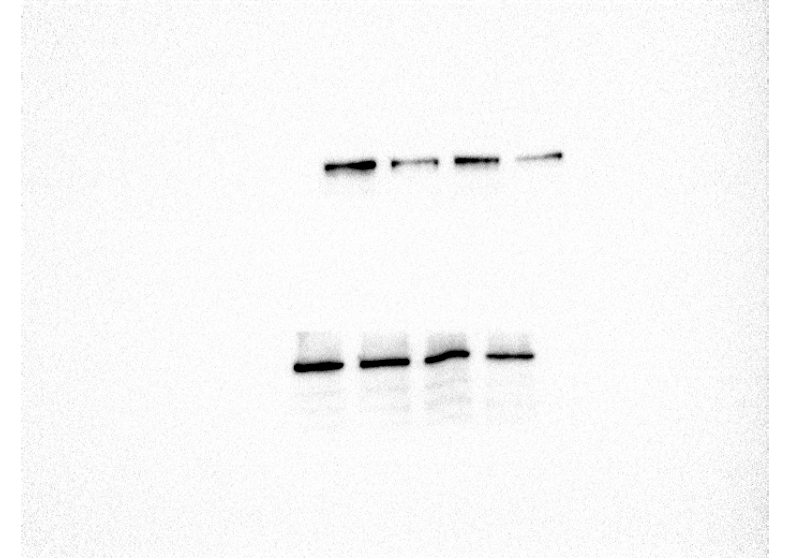

Supplement: Figure 3—source data 2. [file elife-90692-fig3-data2.zip › Figure 3 - Source data 2. Raw unedited membranes for Figure 3/Fig 3C_B-catenin.tif]

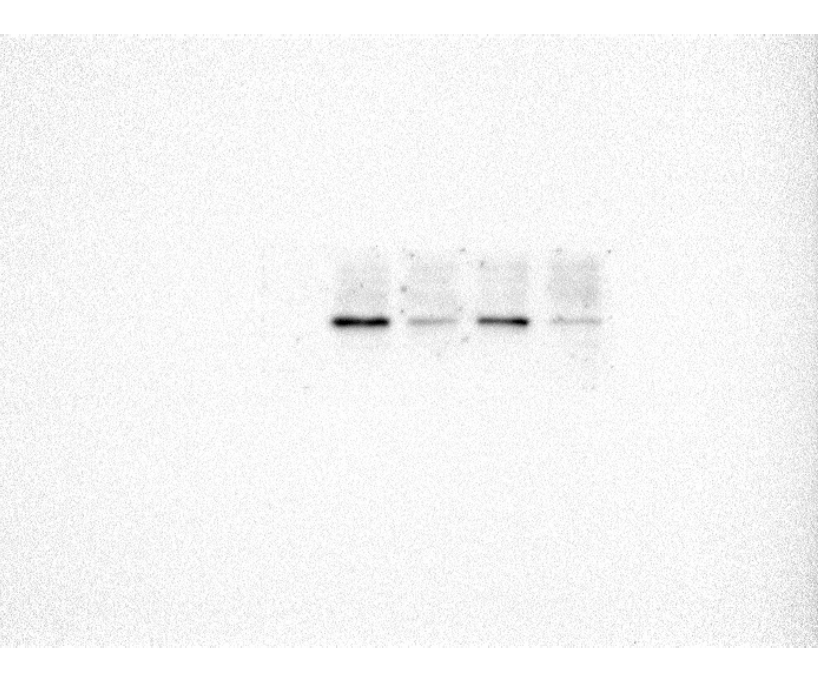

Supplement: Figure 3—source data 2. [file elife-90692-fig3-data2.zip › Figure 3 - Source data 2. Raw unedited membranes for Figure 3/Fig 3C_Arpin.tif]

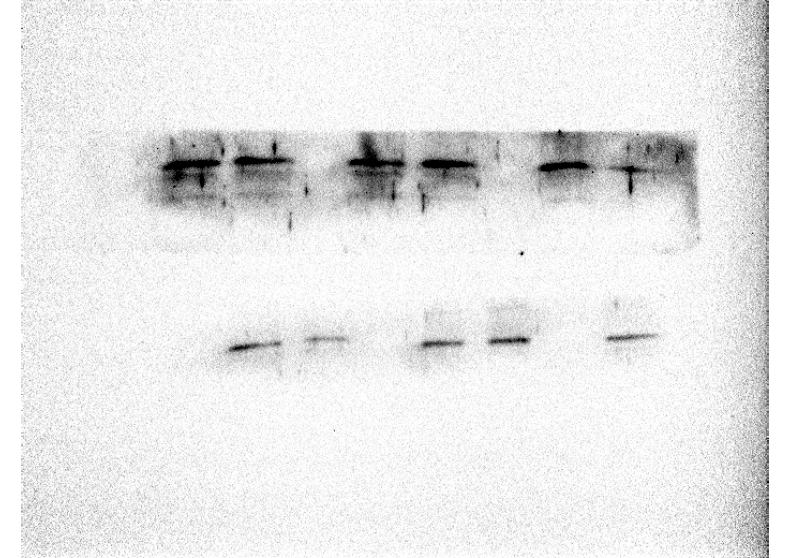

Supplement: Figure 3—source data 2. [file elife-90692-fig3-data2.zip › Figure 3 - Source data 2. Raw unedited membranes for Figure 3/Fig 3A_arpC5.tif]

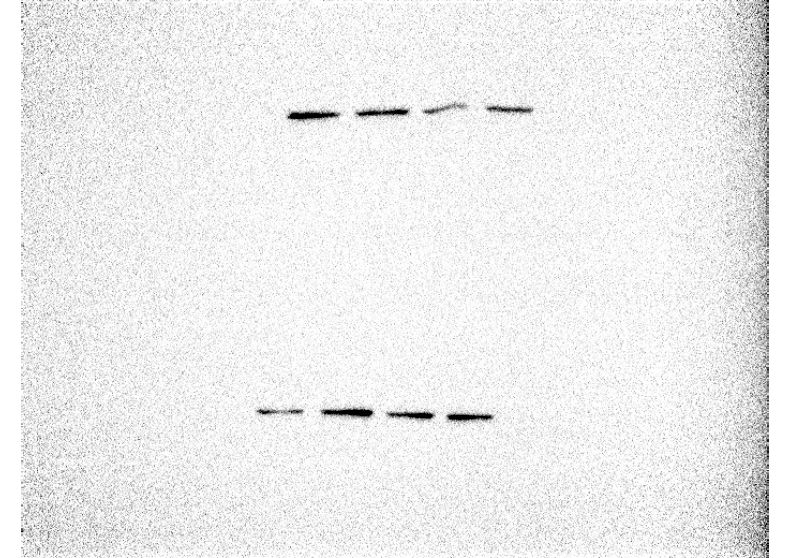

Supplement: Figure 3—source data 2. [file elife-90692-fig3-data2.zip › Figure 3 - Source data 2. Raw unedited membranes for Figure 3/Fig 3C_Gamma Tubulin.tif]

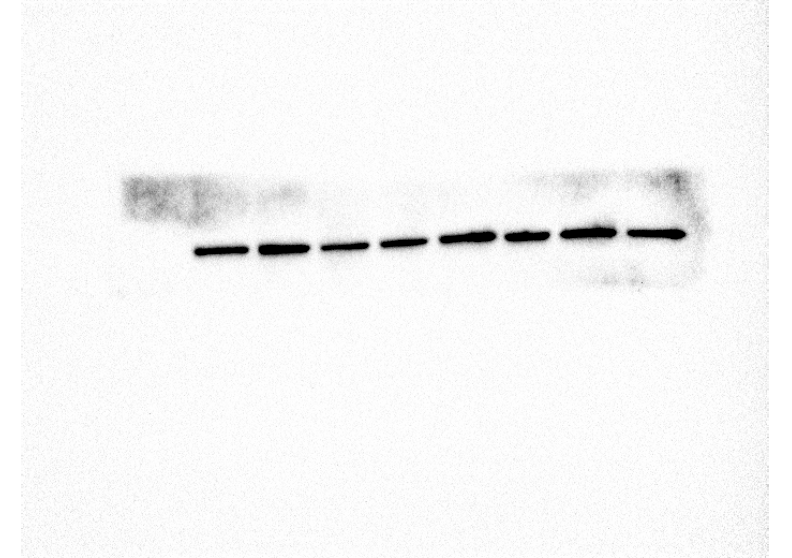

Supplement: Figure 3—source data 2. [file elife-90692-fig3-data2.zip › Figure 3 - Source data 2. Raw unedited membranes for Figure 3/Fig 3A_Gamma Tubulin.tif]

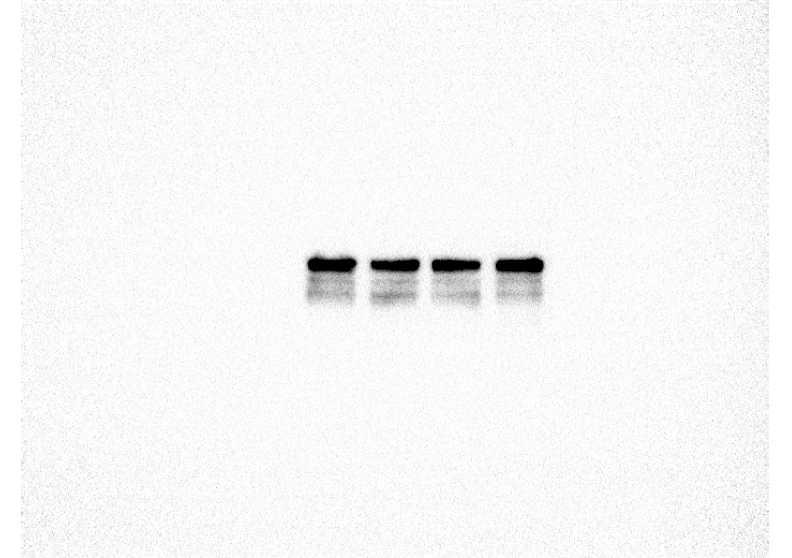

Supplement: Figure 3—source data 2. [file elife-90692-fig3-data2.zip › Figure 3 - Source data 2. Raw unedited membranes for Figure 3/Fig 3C_VE-Cadherin.tif]

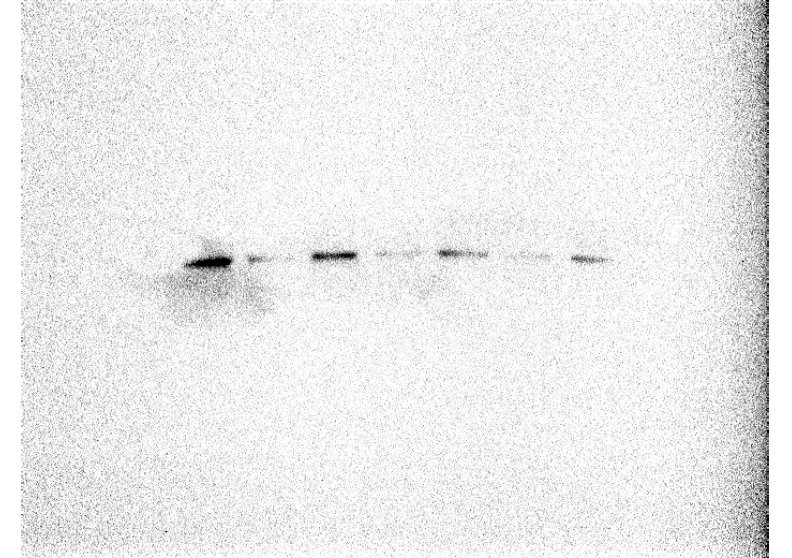

Supplement: Figure 3—source data 2. [file elife-90692-fig3-data2.zip › Figure 3 - Source data 2. Raw unedited membranes for Figure 3/Fig 3D_Arpin.tif]

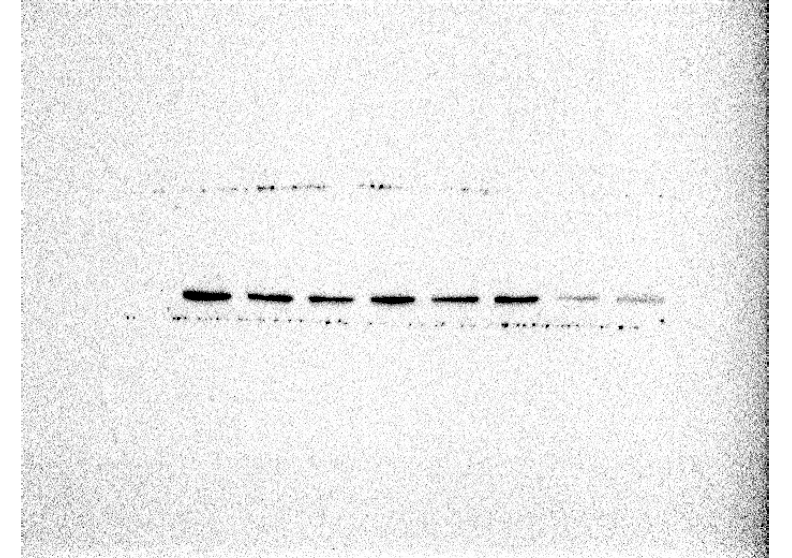

Supplement: Figure 3—source data 2. [file elife-90692-fig3-data2.zip › Figure 3 - Source data 2. Raw unedited membranes for Figure 3/Fig 3D_Gamma Tubulin.tif]

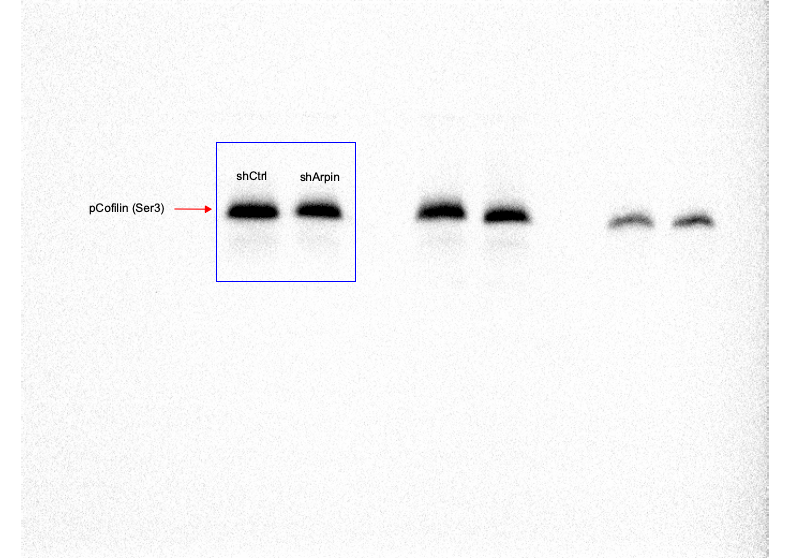

Supplement: Figure 4—source data 1. [file elife-90692-fig4-data1.zip › Figure 4 - Source data 1. Uncropped and labelled membranes for Figure 4/Fig 4D_p-Cofilin (Ser3)_Labelled.tif]

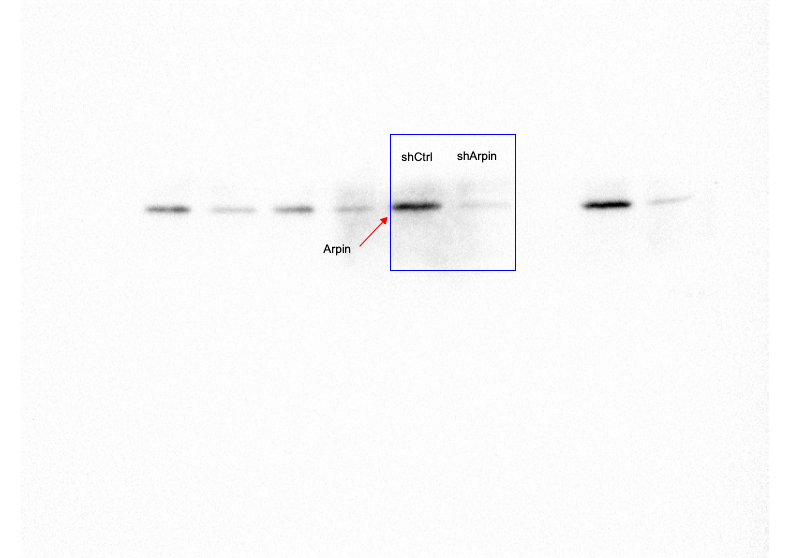

Supplement: Figure 4—source data 1. [file elife-90692-fig4-data1.zip › Figure 4 - Source data 1. Uncropped and labelled membranes for Figure 4/Fig 4C_Arpin_Labelled.tif]

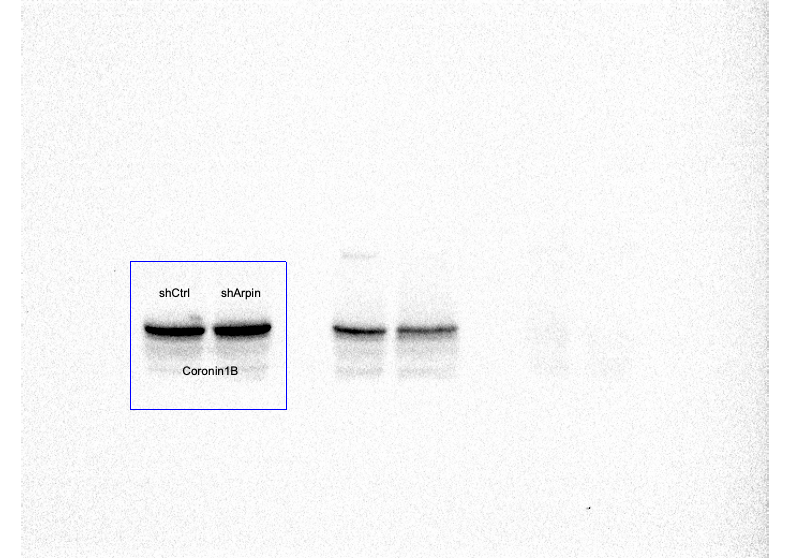

Supplement: Figure 4—source data 1. [file elife-90692-fig4-data1.zip › Figure 4 - Source data 1. Uncropped and labelled membranes for Figure 4/Fig 4D_Coronin1B_Labelled.tif]

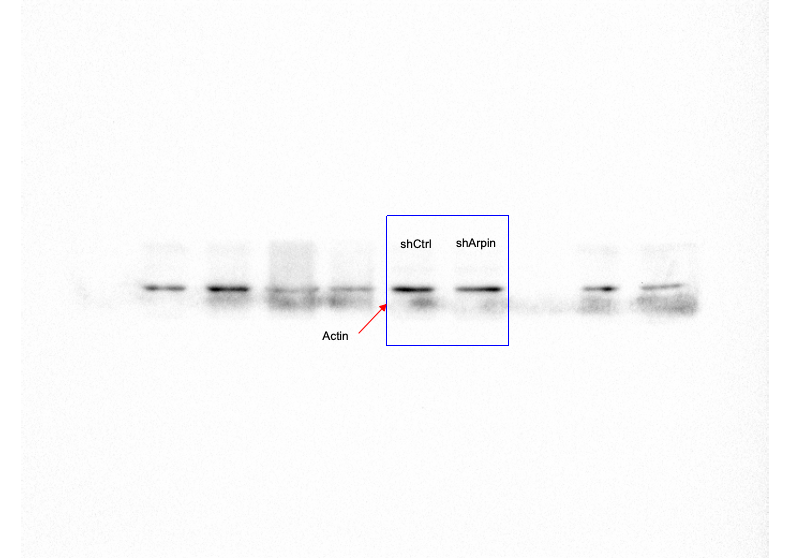

Supplement: Figure 4—source data 1. [file elife-90692-fig4-data1.zip › Figure 4 - Source data 1. Uncropped and labelled membranes for Figure 4/Fig 4C_Actin_Labelled.tif]

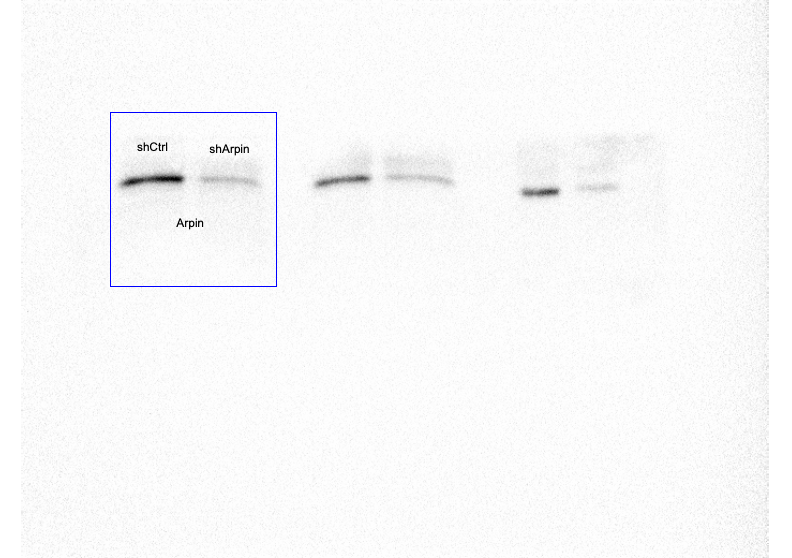

Supplement: Figure 4—source data 1. [file elife-90692-fig4-data1.zip › Figure 4 - Source data 1. Uncropped and labelled membranes for Figure 4/Fig 4D_Arpin_Labelled.tif]

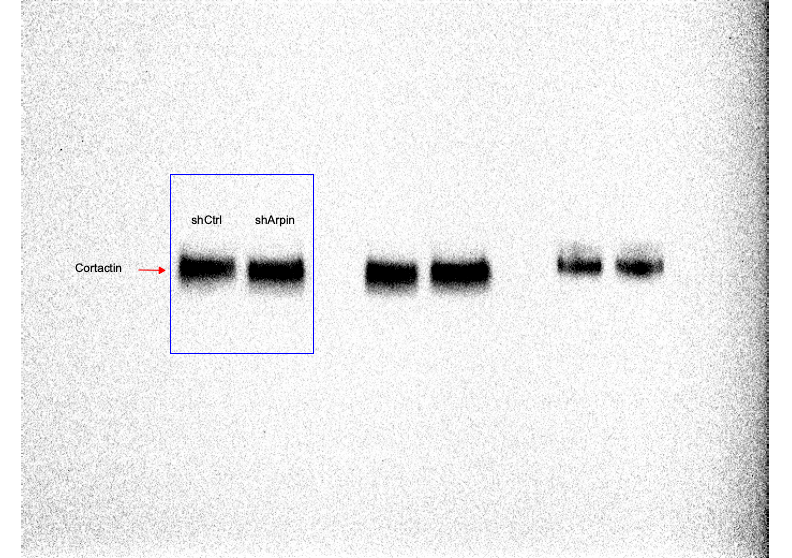

Supplement: Figure 4—source data 1. [file elife-90692-fig4-data1.zip › Figure 4 - Source data 1. Uncropped and labelled membranes for Figure 4/Fig 4D_Cortactin_Labelled.tif]

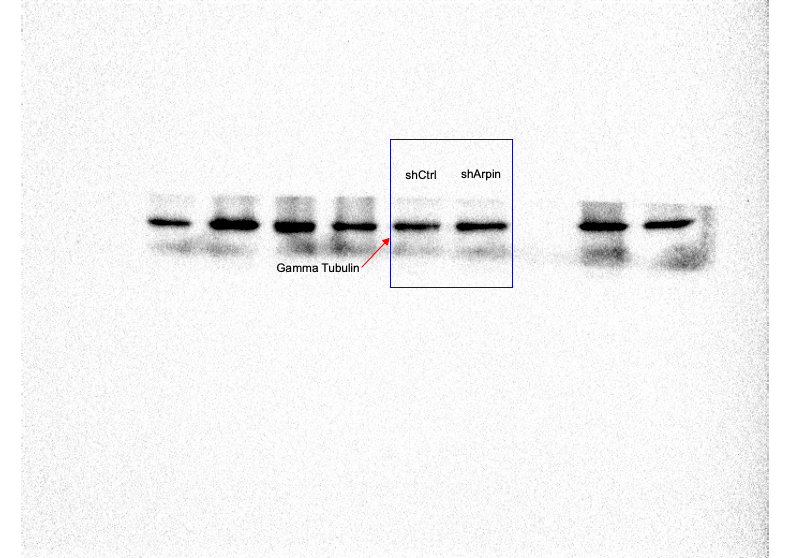

Supplement: Figure 4—source data 1. [file elife-90692-fig4-data1.zip › Figure 4 - Source data 1. Uncropped and labelled membranes for Figure 4/Fig 4C_Gamma Tubulin_Labelled.tif]

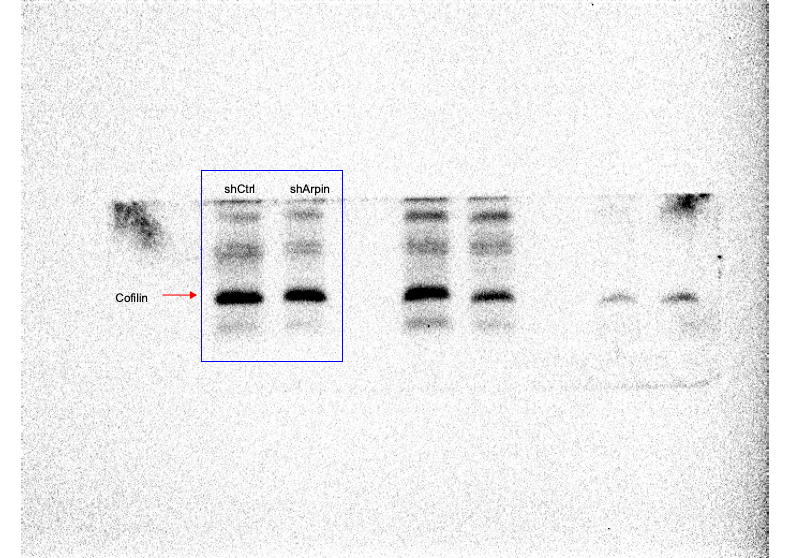

Supplement: Figure 4—source data 1. [file elife-90692-fig4-data1.zip › Figure 4 - Source data 1. Uncropped and labelled membranes for Figure 4/Fig 4D_Cofilin_Labelled.tif]

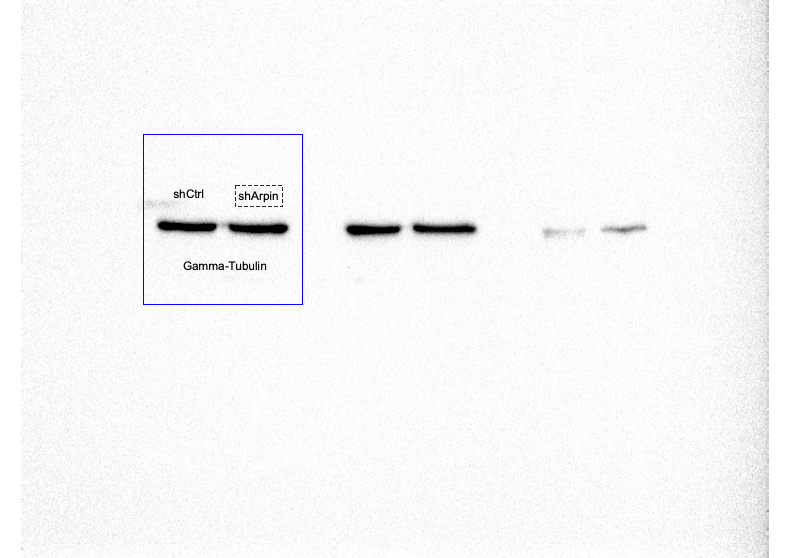

Supplement: Figure 4—source data 1. [file elife-90692-fig4-data1.zip › Figure 4 - Source data 1. Uncropped and labelled membranes for Figure 4/Fig 4D_Gamma Tubulin_Labelled.tif]

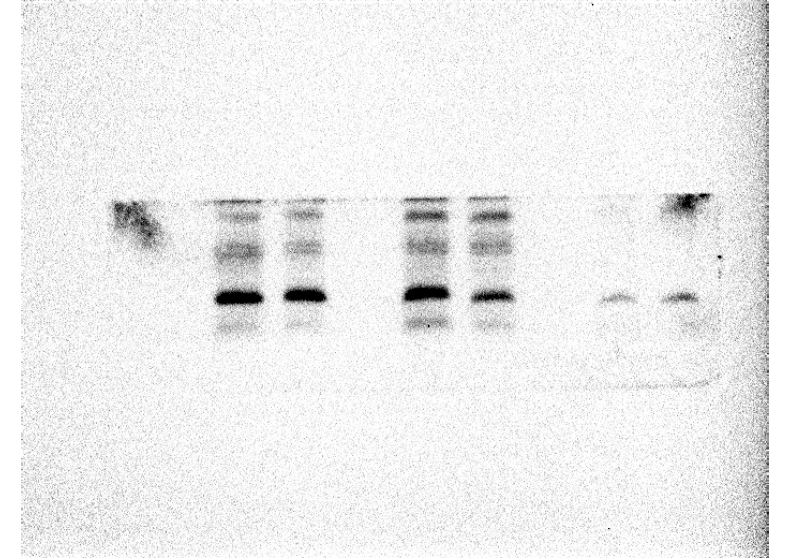

Supplement: Figure 4—source data 2. [file elife-90692-fig4-data2.zip › Figure 4 - Source data 2 - Raw unedited membranes for Figure 4/Fig 4D_Cofilin.tif]

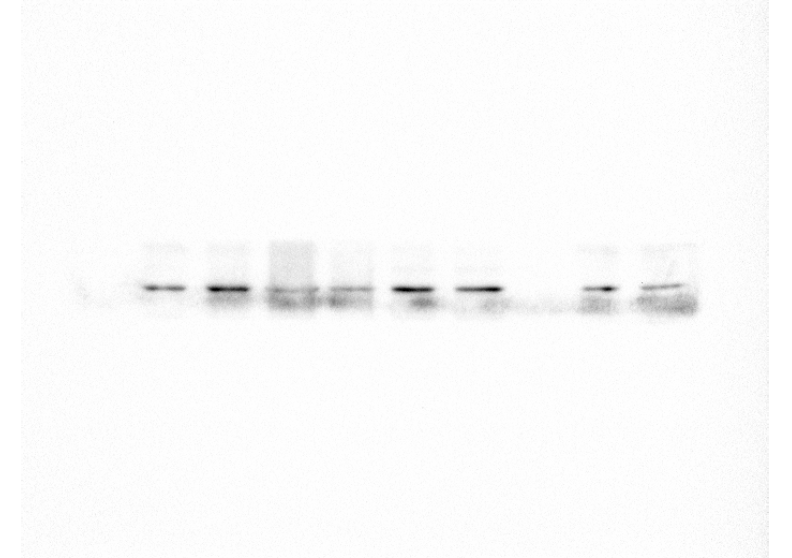

Supplement: Figure 4—source data 2. [file elife-90692-fig4-data2.zip › Figure 4 - Source data 2 - Raw unedited membranes for Figure 4/Fig 4C_Actin.tif]

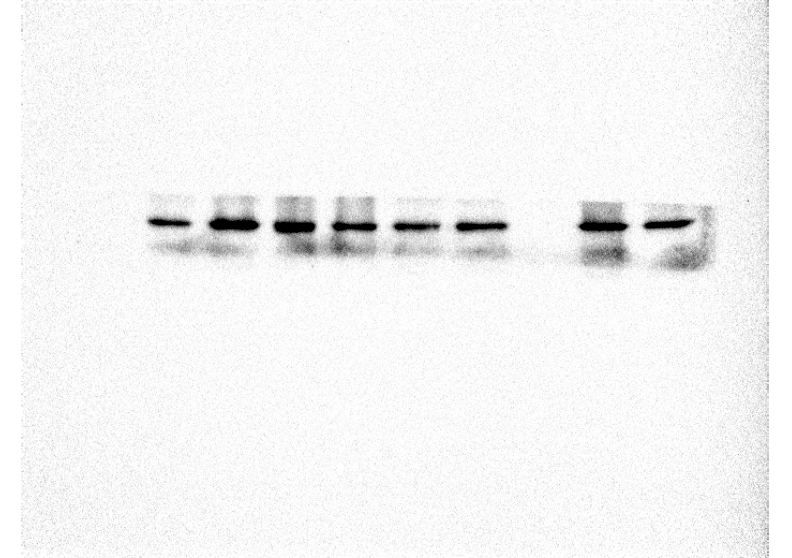

Supplement: Figure 4—source data 2. [file elife-90692-fig4-data2.zip › Figure 4 - Source data 2 - Raw unedited membranes for Figure 4/Fig 4C_Gamma Tubulin.tif]

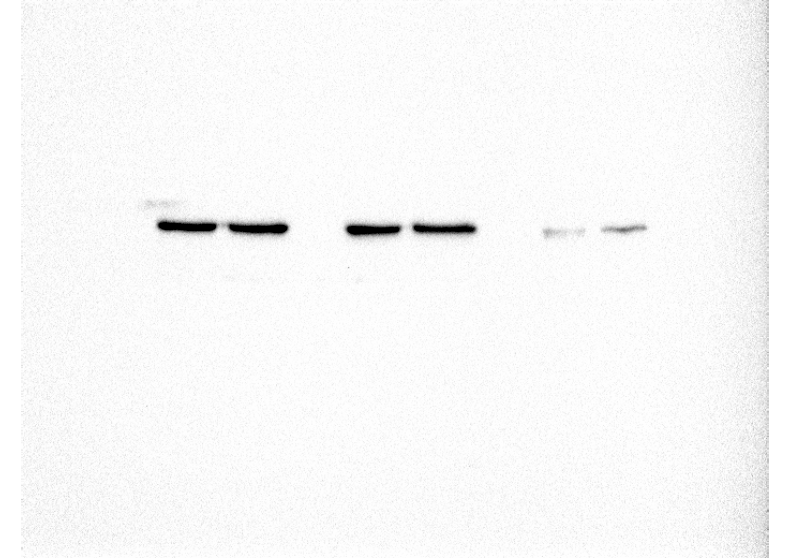

Supplement: Figure 4—source data 2. [file elife-90692-fig4-data2.zip › Figure 4 - Source data 2 - Raw unedited membranes for Figure 4/Fig 4D_Gamma Tubulin.tif]

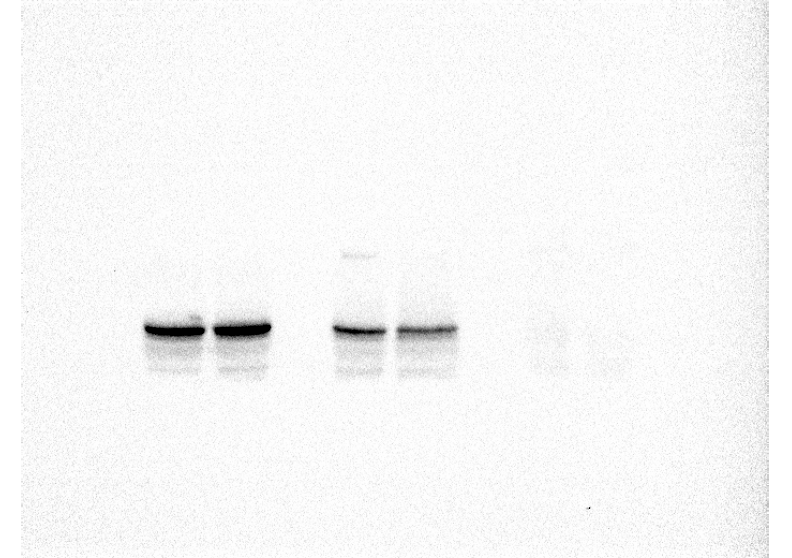

Supplement: Figure 4—source data 2. [file elife-90692-fig4-data2.zip › Figure 4 - Source data 2 - Raw unedited membranes for Figure 4/Fig 4D_Coronin1B.tif]

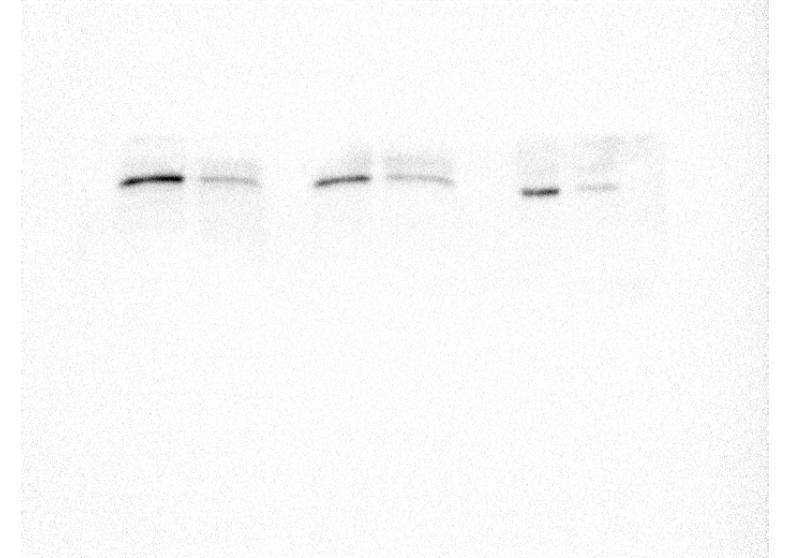

Supplement: Figure 4—source data 2. [file elife-90692-fig4-data2.zip › Figure 4 - Source data 2 - Raw unedited membranes for Figure 4/Fig 4D_Arpin.tif]

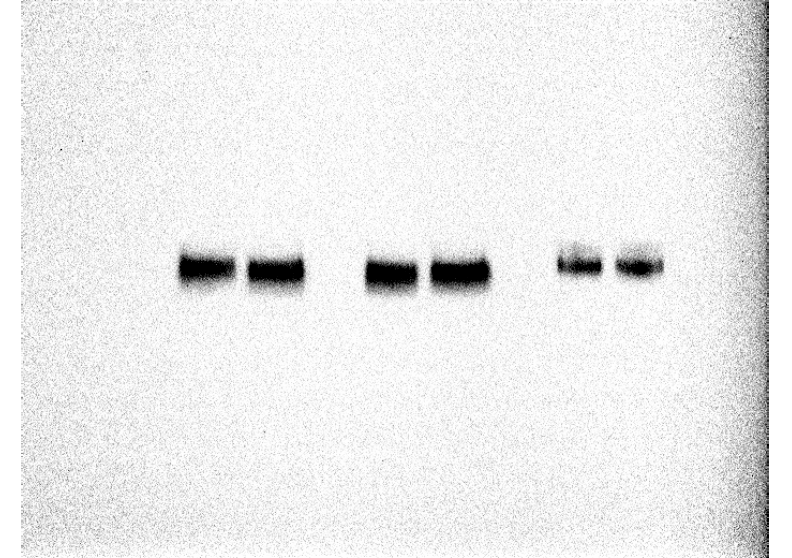

Supplement: Figure 4—source data 2. [file elife-90692-fig4-data2.zip › Figure 4 - Source data 2 - Raw unedited membranes for Figure 4/Fig 4D_Cortactin.tif]

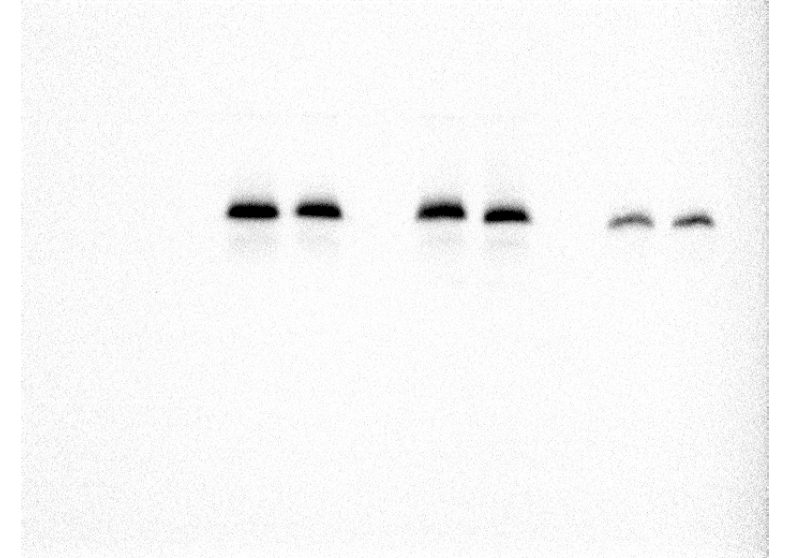

Supplement: Figure 4—source data 2. [file elife-90692-fig4-data2.zip › Figure 4 - Source data 2 - Raw unedited membranes for Figure 4/Fig 4D_p-Cofilin (Ser3).tif]

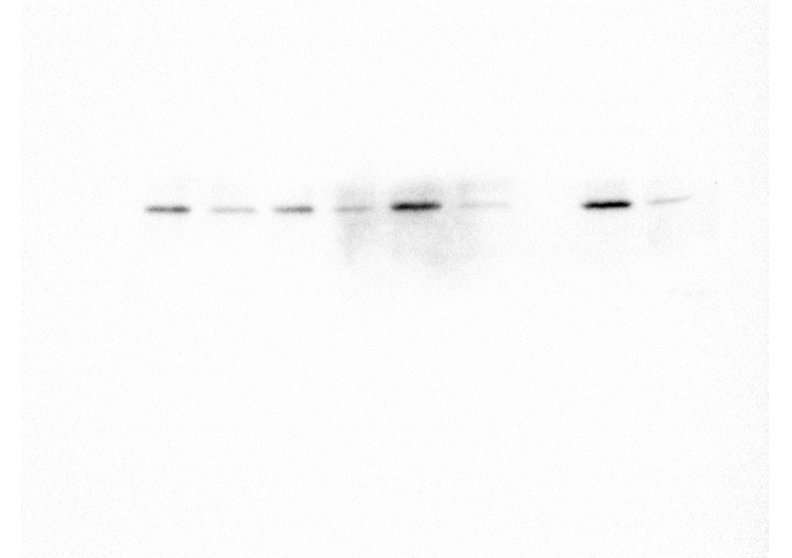

Supplement: Figure 4—source data 2. [file elife-90692-fig4-data2.zip › Figure 4 - Source data 2 - Raw unedited membranes for Figure 4/Fig 4C_Arpin.tif]

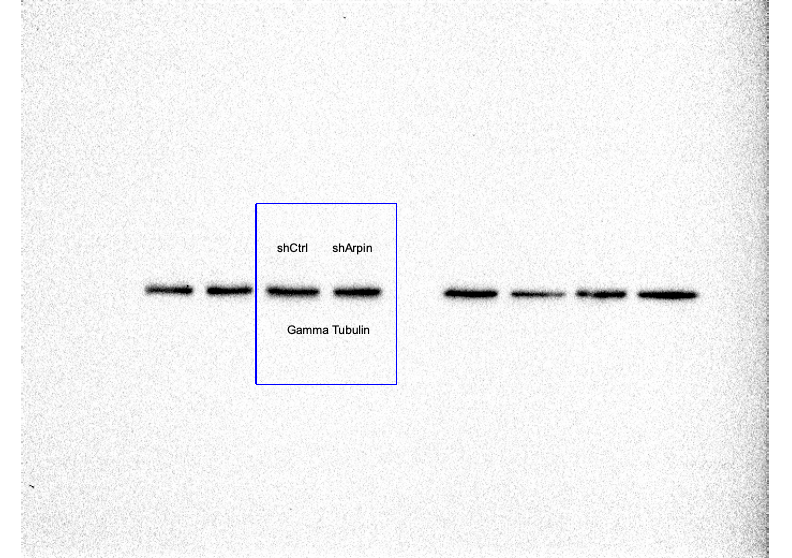

Supplement: Figure 6—source data 1. [file elife-90692-fig6-data1.zip › Figure 6 - Source data 1. Uncropped and labelled membranes for Figure 6/Fig 6B_Gamma Tubulin_Labelled.tif]

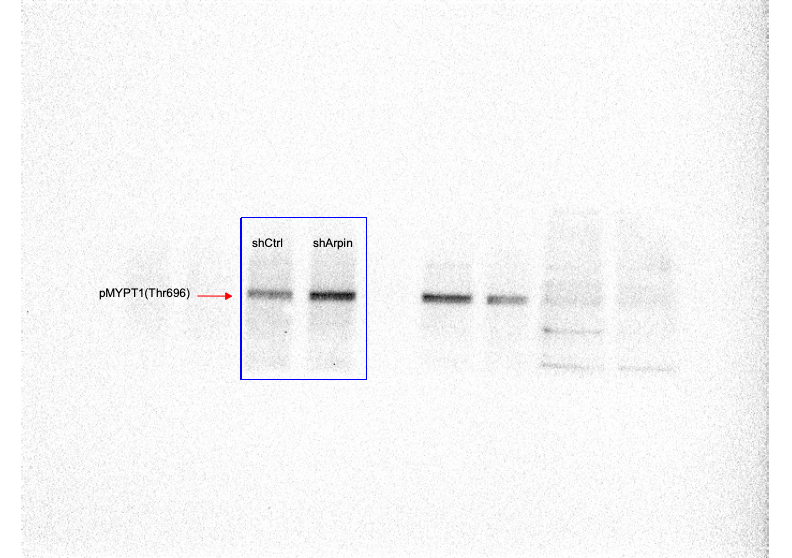

Supplement: Figure 6—source data 1. [file elife-90692-fig6-data1.zip › Figure 6 - Source data 1. Uncropped and labelled membranes for Figure 6/Fig 6B_pMYPT1(Thr696)_Labelled.tif]

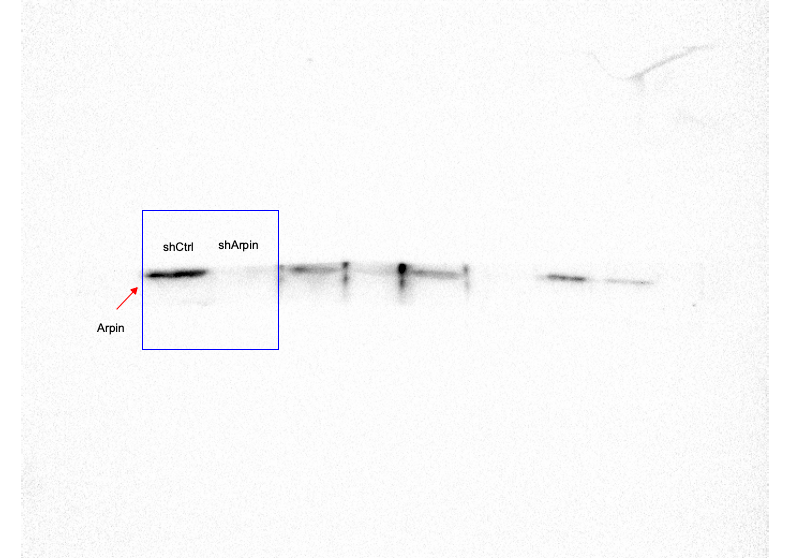

Supplement: Figure 6—source data 1. [file elife-90692-fig6-data1.zip › Figure 6 - Source data 1. Uncropped and labelled membranes for Figure 6/Fig 6C_Arpin_Labelled.tif]

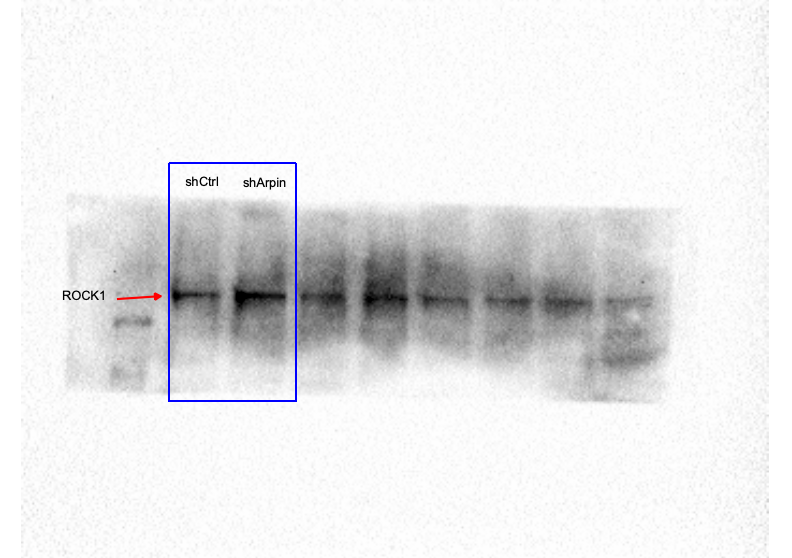

Supplement: Figure 6—source data 1. [file elife-90692-fig6-data1.zip › Figure 6 - Source data 1. Uncropped and labelled membranes for Figure 6/Fig 6C_ROCK1_Labelled.tif]

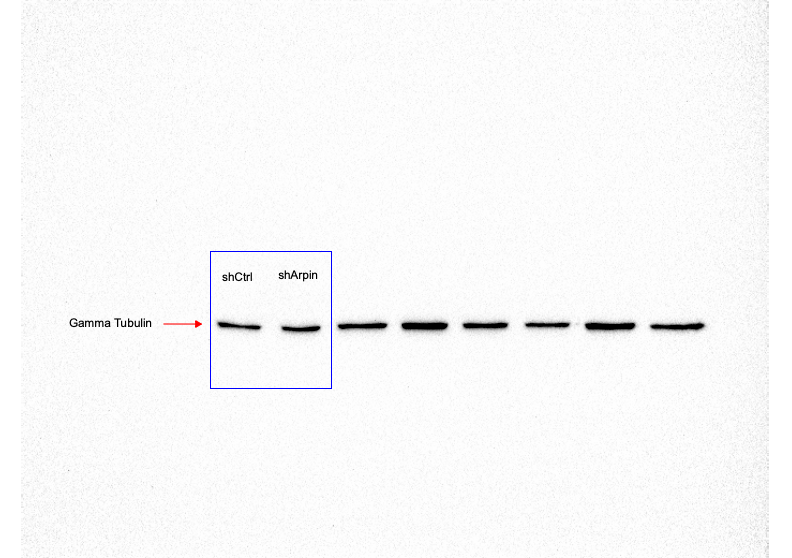

Supplement: Figure 6—source data 1. [file elife-90692-fig6-data1.zip › Figure 6 - Source data 1. Uncropped and labelled membranes for Figure 6/Fig 6C_Gamma Tubulin_Labelled.tif]

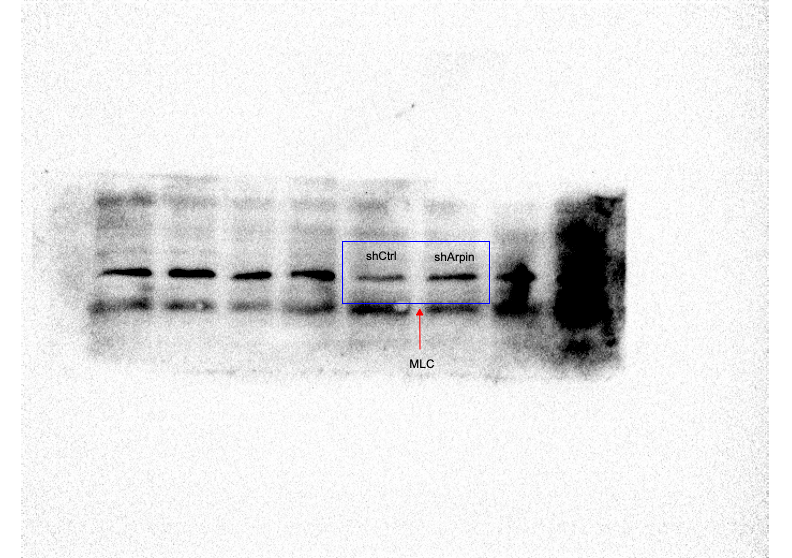

Supplement: Figure 6—source data 1. [file elife-90692-fig6-data1.zip › Figure 6 - Source data 1. Uncropped and labelled membranes for Figure 6/Fig 6A_MLC_Labelled.tif]

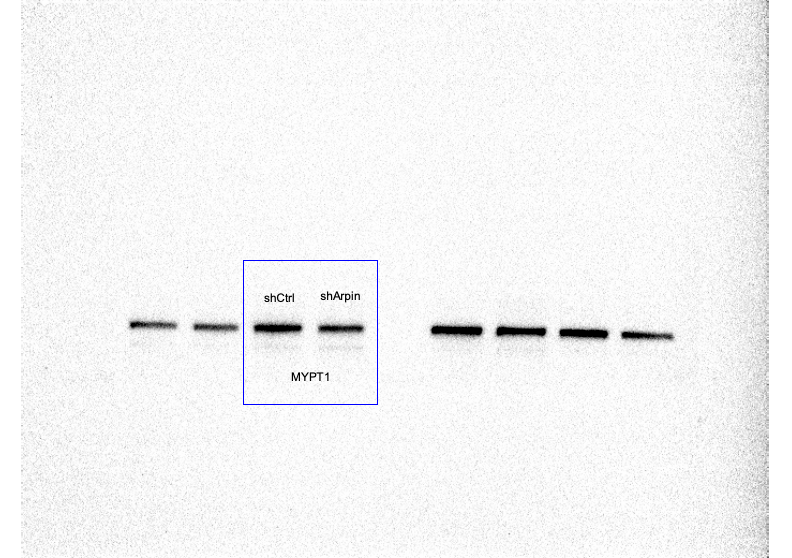

Supplement: Figure 6—source data 1. [file elife-90692-fig6-data1.zip › Figure 6 - Source data 1. Uncropped and labelled membranes for Figure 6/Fig 6B_MYPT1_Labelled.tif]

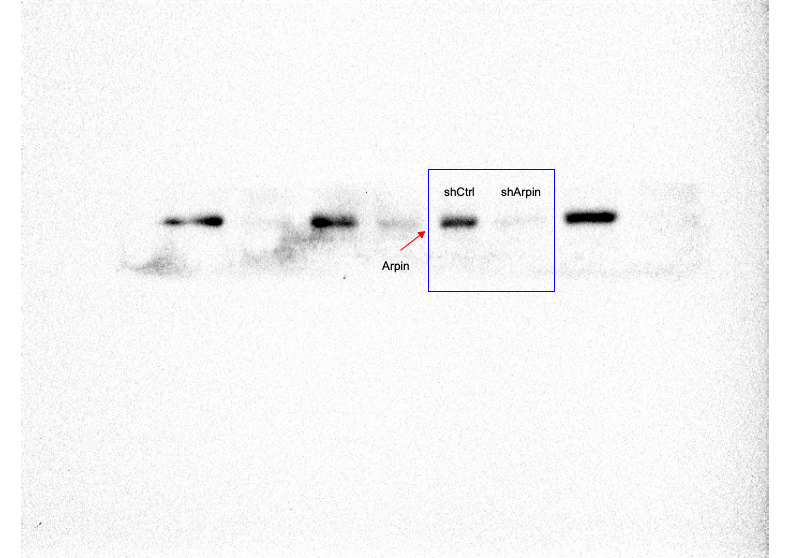

Supplement: Figure 6—source data 1. [file elife-90692-fig6-data1.zip › Figure 6 - Source data 1. Uncropped and labelled membranes for Figure 6/Fig 6A_Arpin_Labelled.tif]

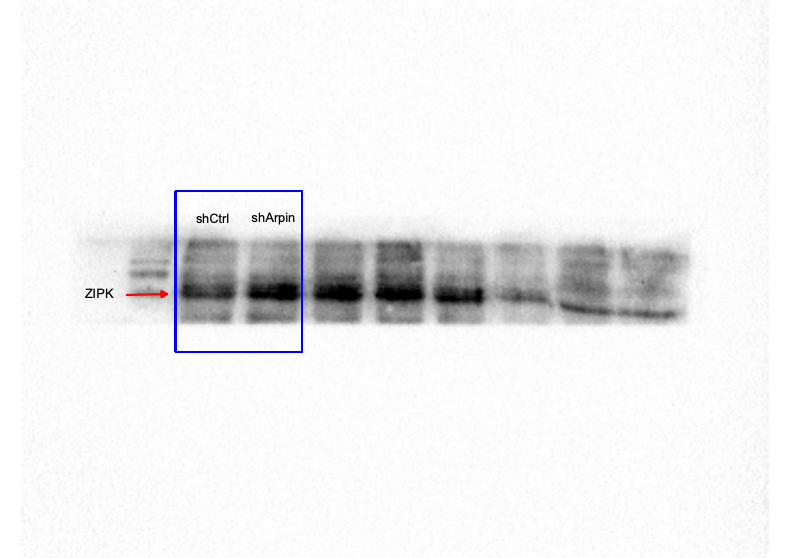

Supplement: Figure 6—source data 1. [file elife-90692-fig6-data1.zip › Figure 6 - Source data 1. Uncropped and labelled membranes for Figure 6/Fig 6C_ZIPK_Labelled.tif]

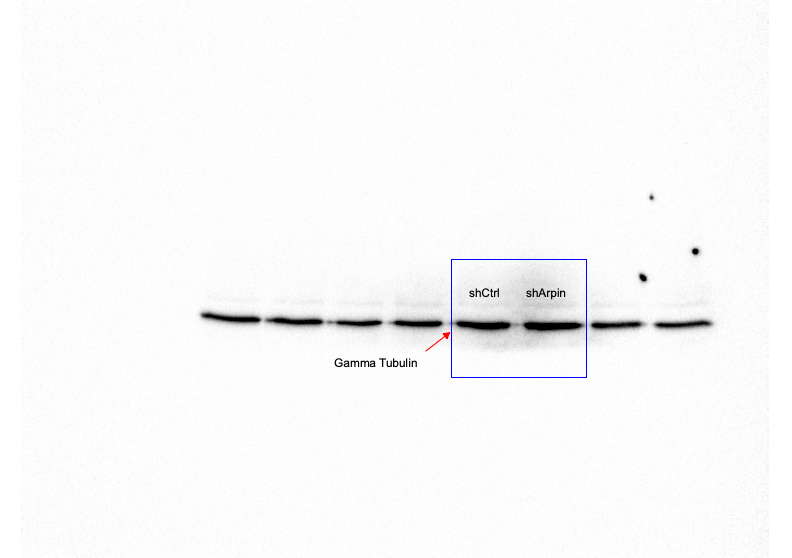

Supplement: Figure 6—source data 1. [file elife-90692-fig6-data1.zip › Figure 6 - Source data 1. Uncropped and labelled membranes for Figure 6/Fig 6A_Gamma Tubulin_Labelled.tif]

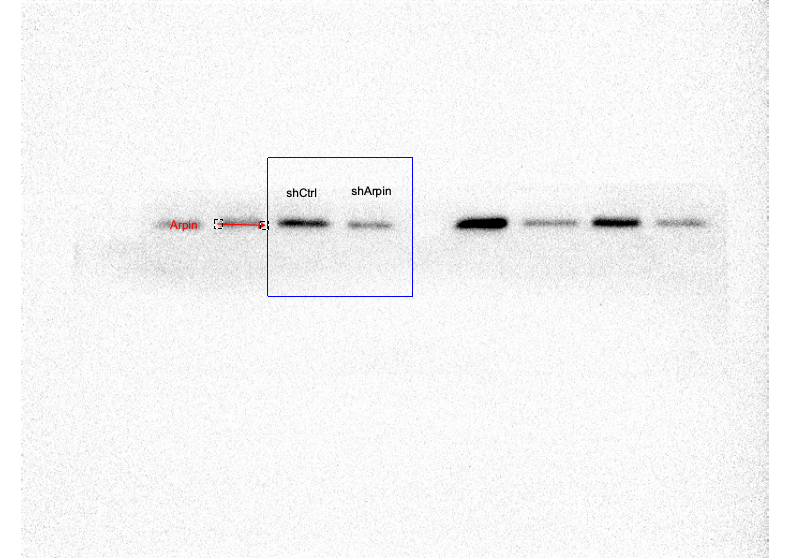

Supplement: Figure 6—source data 1. [file elife-90692-fig6-data1.zip › Figure 6 - Source data 1. Uncropped and labelled membranes for Figure 6/Fig 6B_Arpin_Labelled.tif]

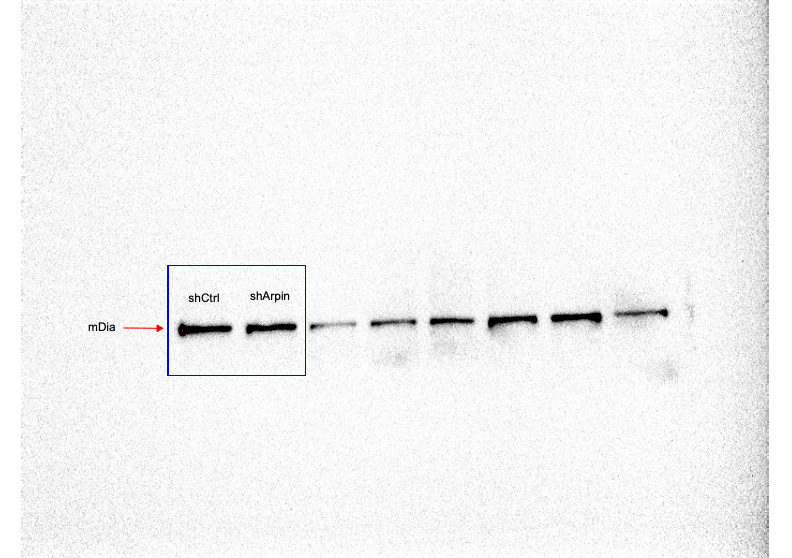

Supplement: Figure 6—source data 1. [file elife-90692-fig6-data1.zip › Figure 6 - Source data 1. Uncropped and labelled membranes for Figure 6/Fig 6C_mDia_Labelled.tif]

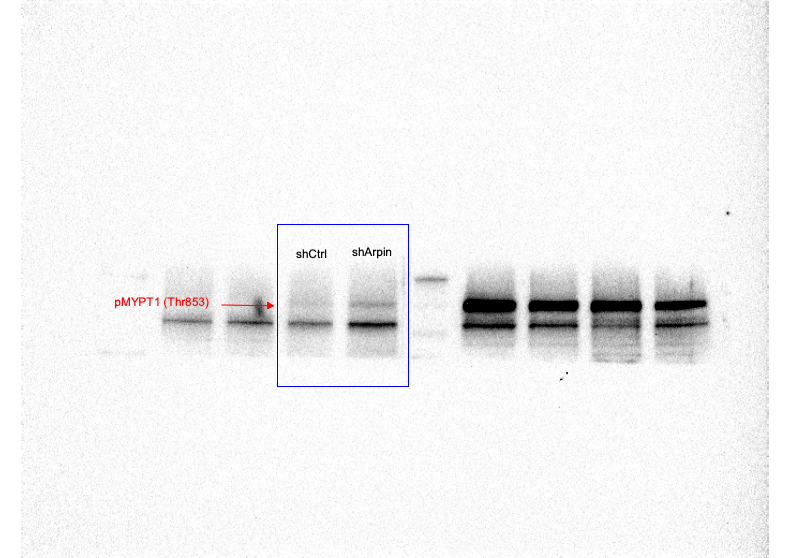

Supplement: Figure 6—source data 1. [file elife-90692-fig6-data1.zip › Figure 6 - Source data 1. Uncropped and labelled membranes for Figure 6/Fig 6B_pMYPT1(Thr853)_Labelled.tif]

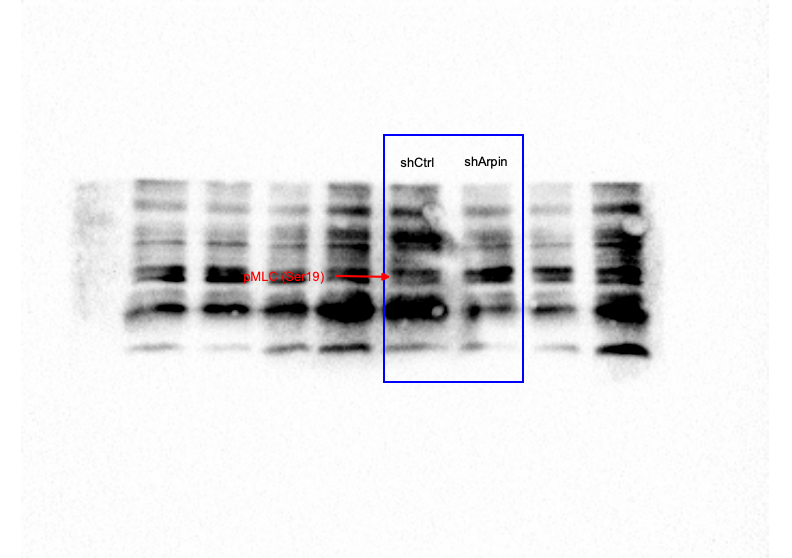

Supplement: Figure 6—source data 1. [file elife-90692-fig6-data1.zip › Figure 6 - Source data 1. Uncropped and labelled membranes for Figure 6/Fig 6A_pMLC (Ser19)_Labelled.tif]

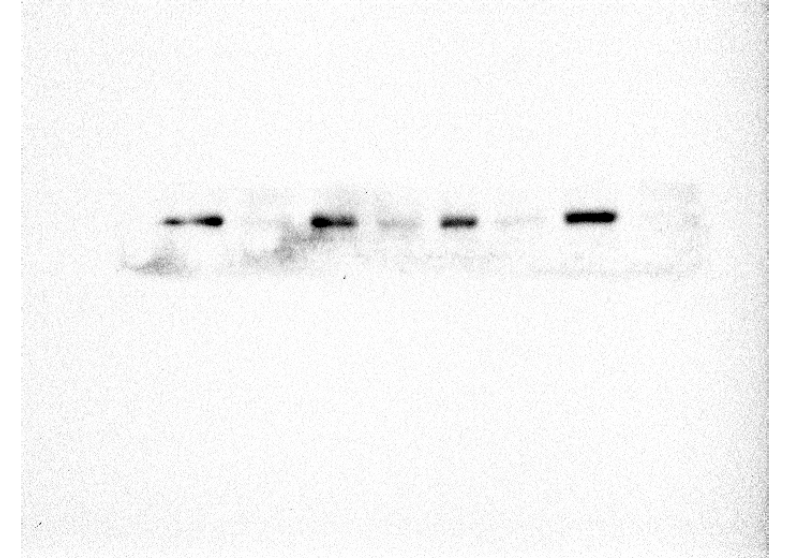

Supplement: Figure 6—source data 2. [file elife-90692-fig6-data2.zip › Figure 6 - Source data 2. Raw unedited membranes for Figure 6/Fig 6A_Arpin.tif]

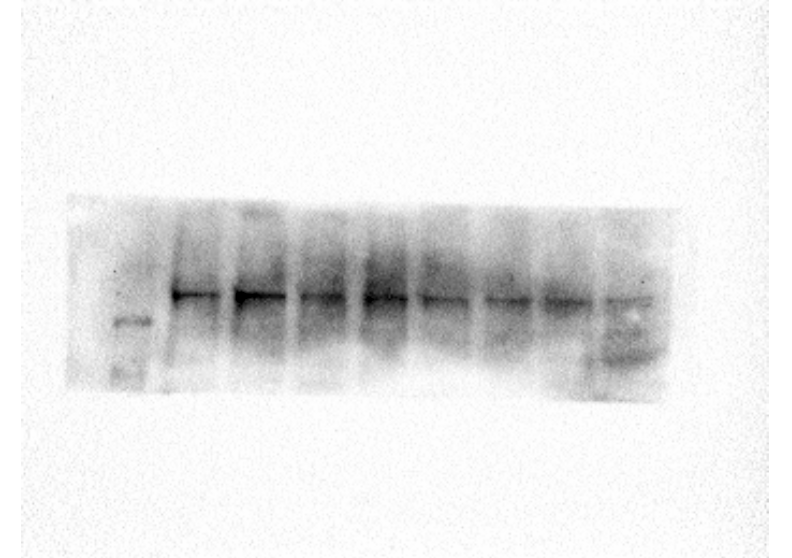

Supplement: Figure 6—source data 2. [file elife-90692-fig6-data2.zip › Figure 6 - Source data 2. Raw unedited membranes for Figure 6/Fig 6C_ROCK1.tif]

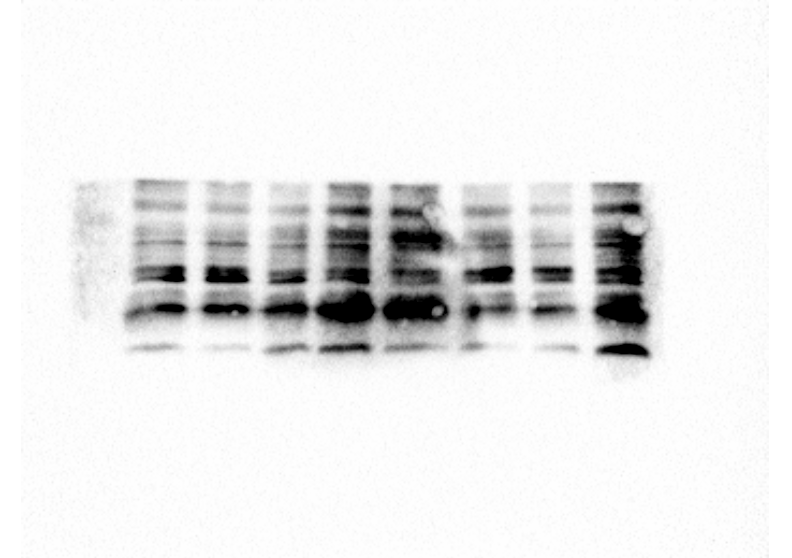

Supplement: Figure 6—source data 2. [file elife-90692-fig6-data2.zip › Figure 6 - Source data 2. Raw unedited membranes for Figure 6/Fig 6A_pMLC (Ser19).tif]

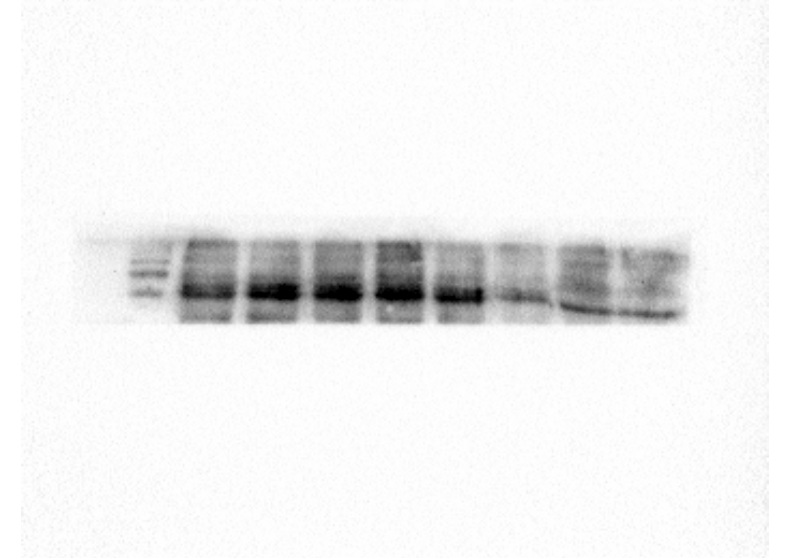

Supplement: Figure 6—source data 2. [file elife-90692-fig6-data2.zip › Figure 6 - Source data 2. Raw unedited membranes for Figure 6/Fig 6C_ZIPK.tif]

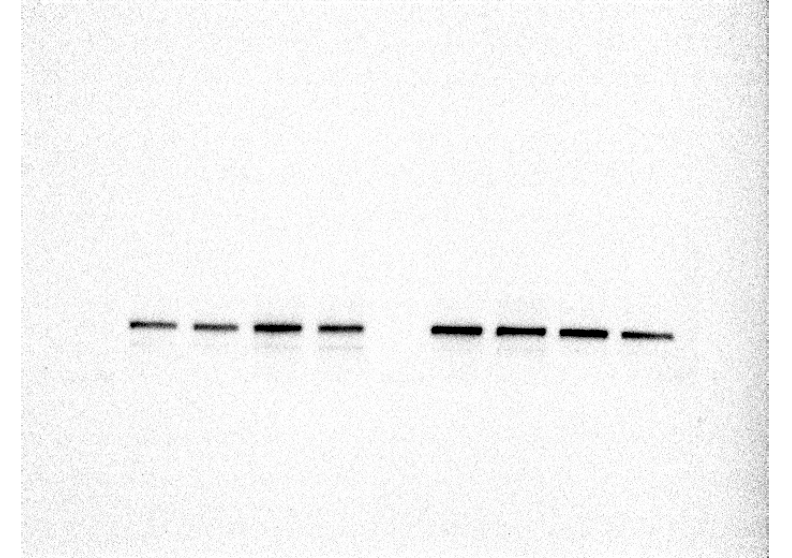

Supplement: Figure 6—source data 2. [file elife-90692-fig6-data2.zip › Figure 6 - Source data 2. Raw unedited membranes for Figure 6/Fig 6B_MYPT1.tif]

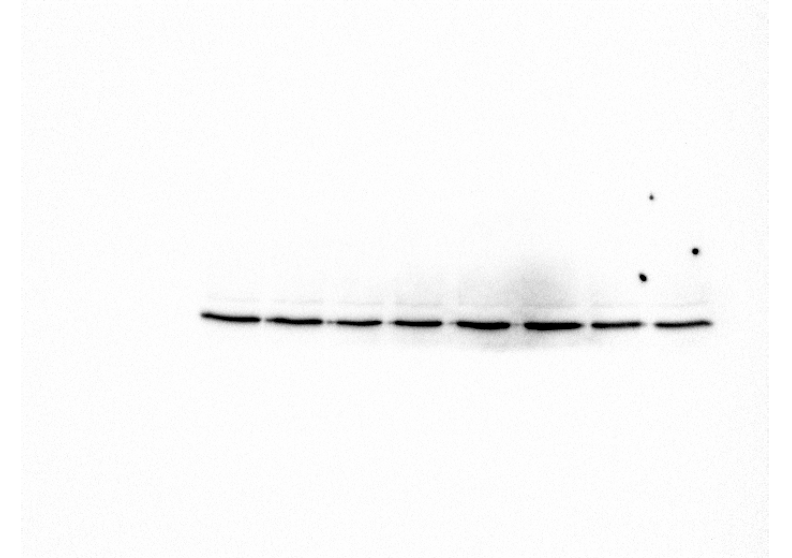

Supplement: Figure 6—source data 2. [file elife-90692-fig6-data2.zip › Figure 6 - Source data 2. Raw unedited membranes for Figure 6/Fig 6A_Gamma Tubulin.tif]

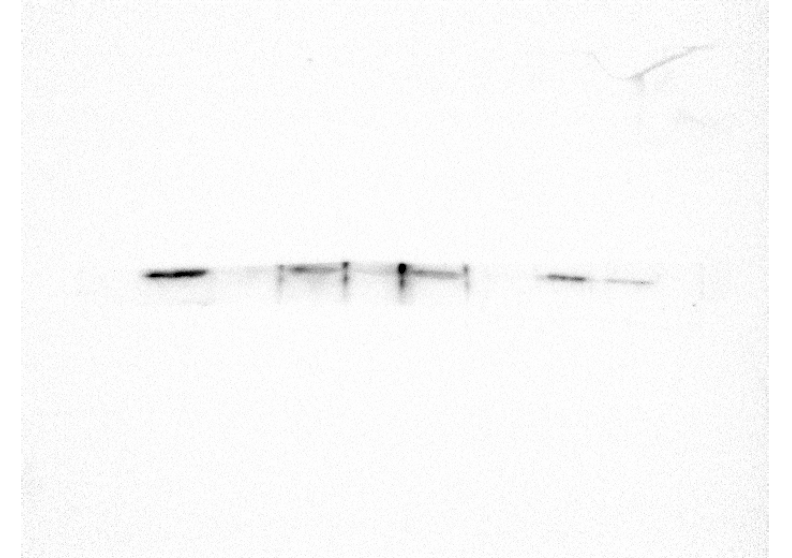

Supplement: Figure 6—source data 2. [file elife-90692-fig6-data2.zip › Figure 6 - Source data 2. Raw unedited membranes for Figure 6/Fig 6C_Arpin.tif]

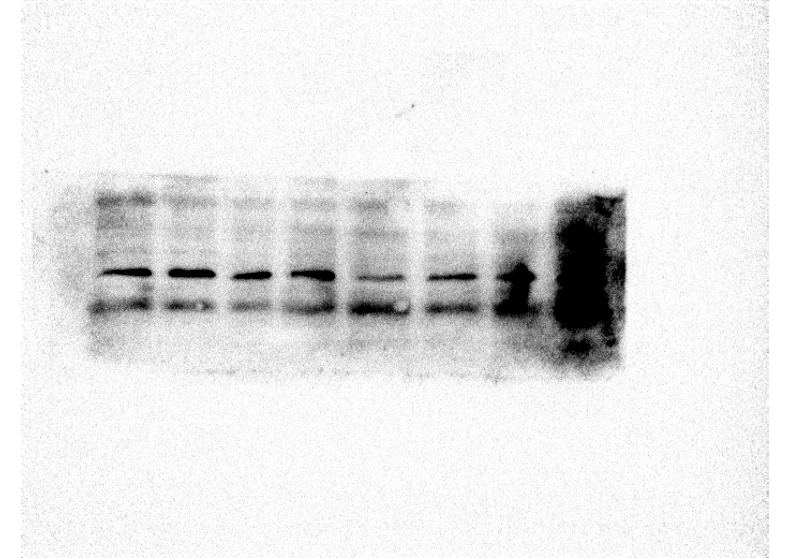

Supplement: Figure 6—source data 2. [file elife-90692-fig6-data2.zip › Figure 6 - Source data 2. Raw unedited membranes for Figure 6/Fig 6A_MLC.tif]

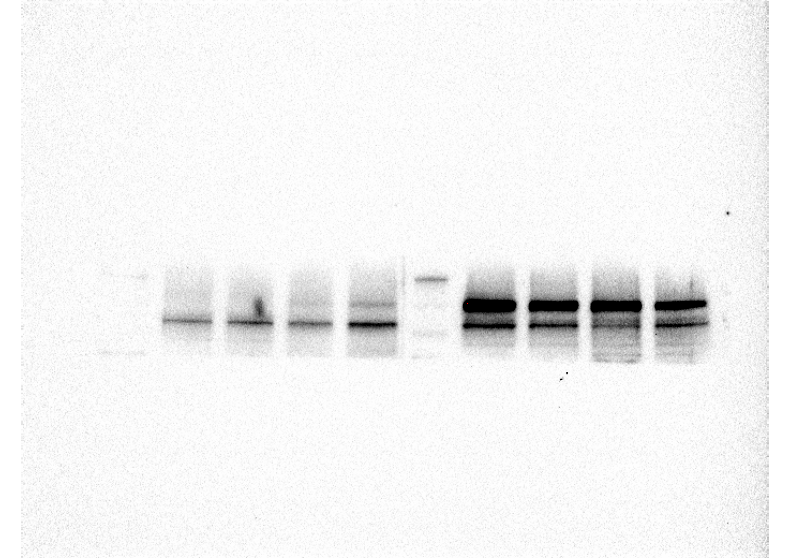

Supplement: Figure 6—source data 2. [file elife-90692-fig6-data2.zip › Figure 6 - Source data 2. Raw unedited membranes for Figure 6/Fig 6B_pMYPT1(Thr853).tif]

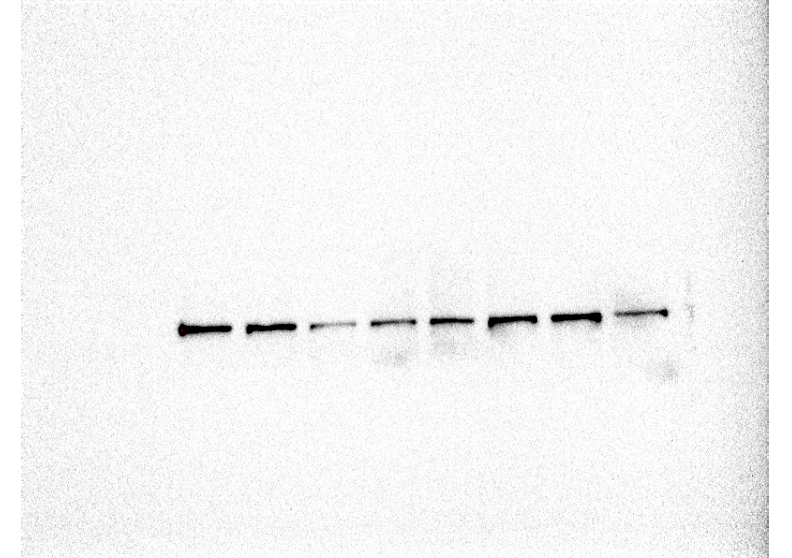

Supplement: Figure 6—source data 2. [file elife-90692-fig6-data2.zip › Figure 6 - Source data 2. Raw unedited membranes for Figure 6/Fig 6C_mDia.tif]

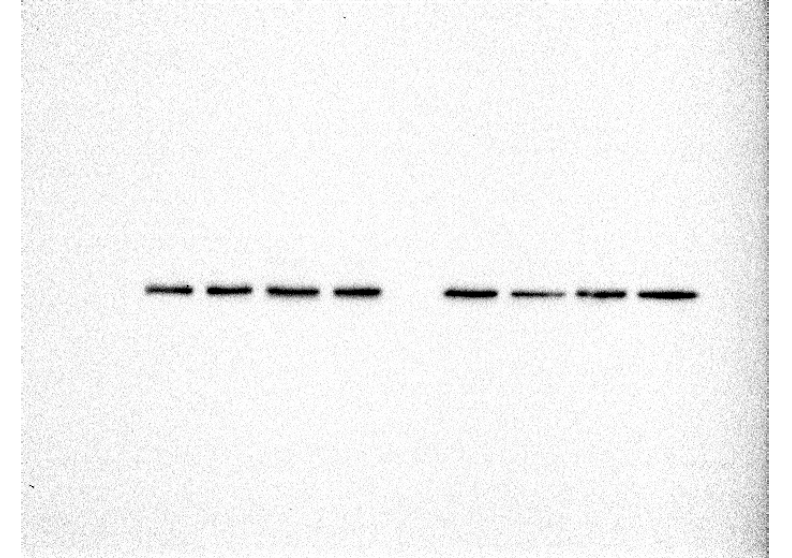

Supplement: Figure 6—source data 2. [file elife-90692-fig6-data2.zip › Figure 6 - Source data 2. Raw unedited membranes for Figure 6/Fig 6B_Gamma Tubulin.tif]

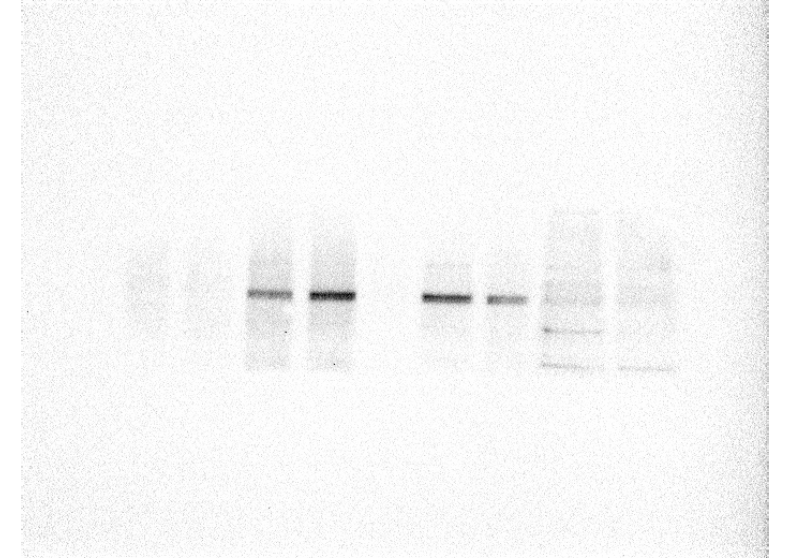

Supplement: Figure 6—source data 2. [file elife-90692-fig6-data2.zip › Figure 6 - Source data 2. Raw unedited membranes for Figure 6/Fig 6B_pMYPT1(Thr696).tif]

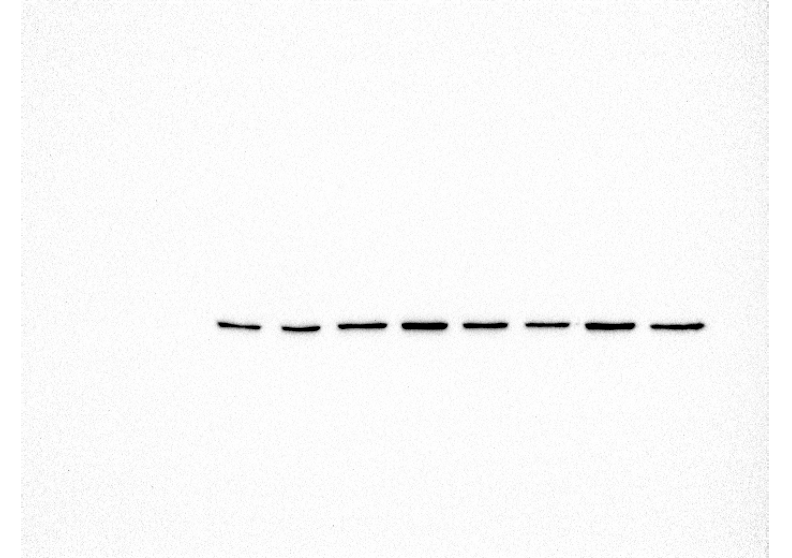

Supplement: Figure 6—source data 2. [file elife-90692-fig6-data2.zip › Figure 6 - Source data 2. Raw unedited membranes for Figure 6/Fig 6C_Gamma Tubulin.tif]

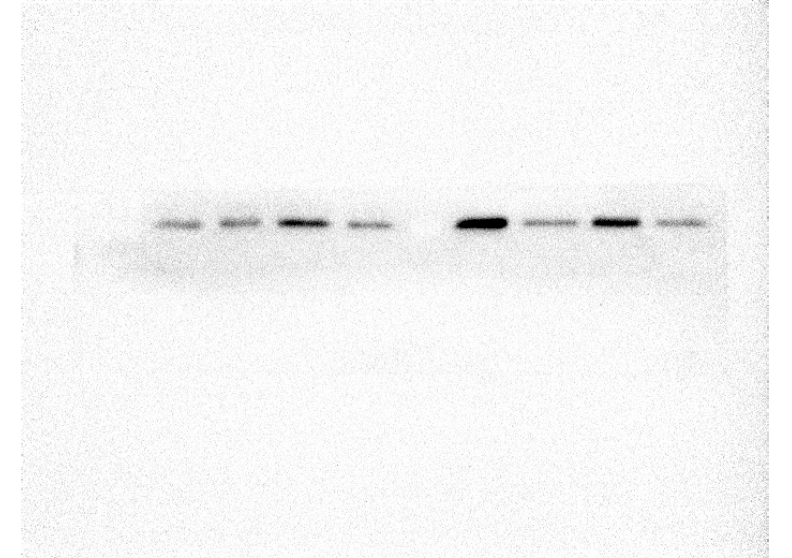

Supplement: Figure 6—source data 2. [file elife-90692-fig6-data2.zip › Figure 6 - Source data 2. Raw unedited membranes for Figure 6/Fig 6B_Arpin.tif]
