# Supplementary figures and images for: Arpin deficiency increases actomyosin contractility and vascular permeability (part 2 of 2)
Source: eLife. 2024 Sep 19;12:RP90692. doi: 10.7554/eLife.90692 (PMC11412691; doi:10.7554/eLife.90692)

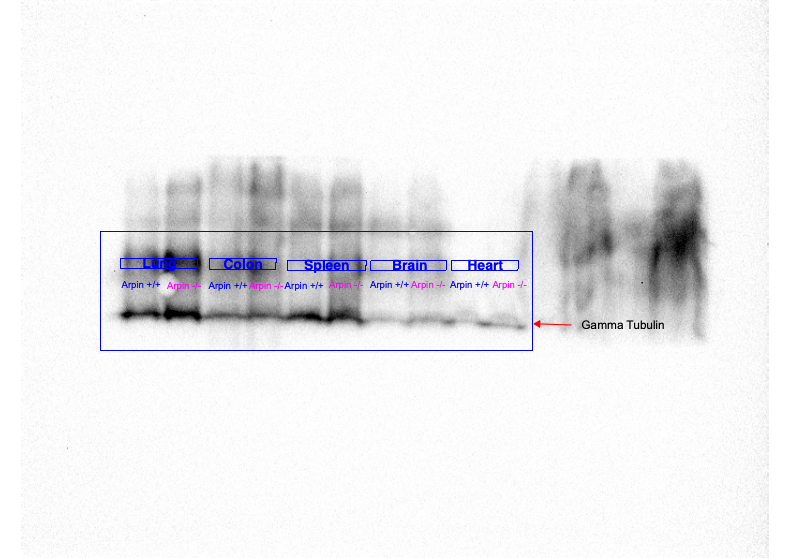

Supplement: Figure 8—source data 1. [file elife-90692-fig8-data1.zip › Figure 8 - Source data 1. Uncropped and labelled membranes for Figure 8/Fig 8C_Gamma Tubulin_Labelled.tif]

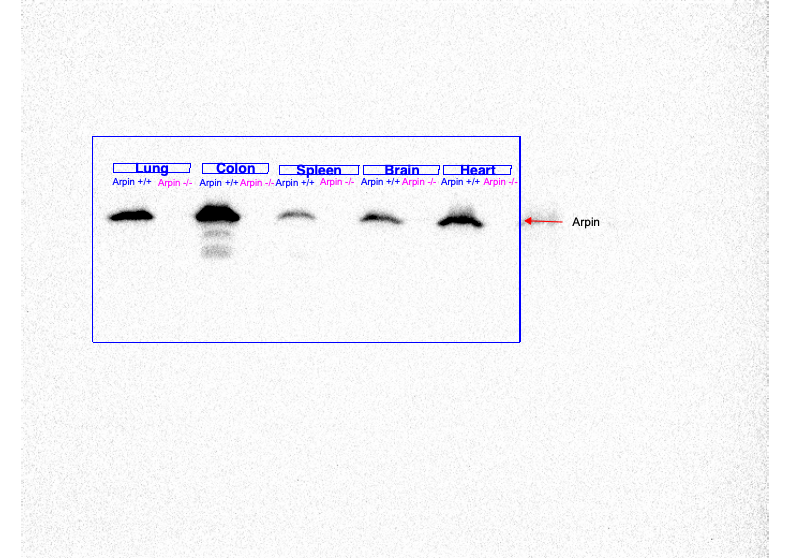

Supplement: Figure 8—source data 1. [file elife-90692-fig8-data1.zip › Figure 8 - Source data 1. Uncropped and labelled membranes for Figure 8/Fig 8C_Arpin_Labelled.tif]

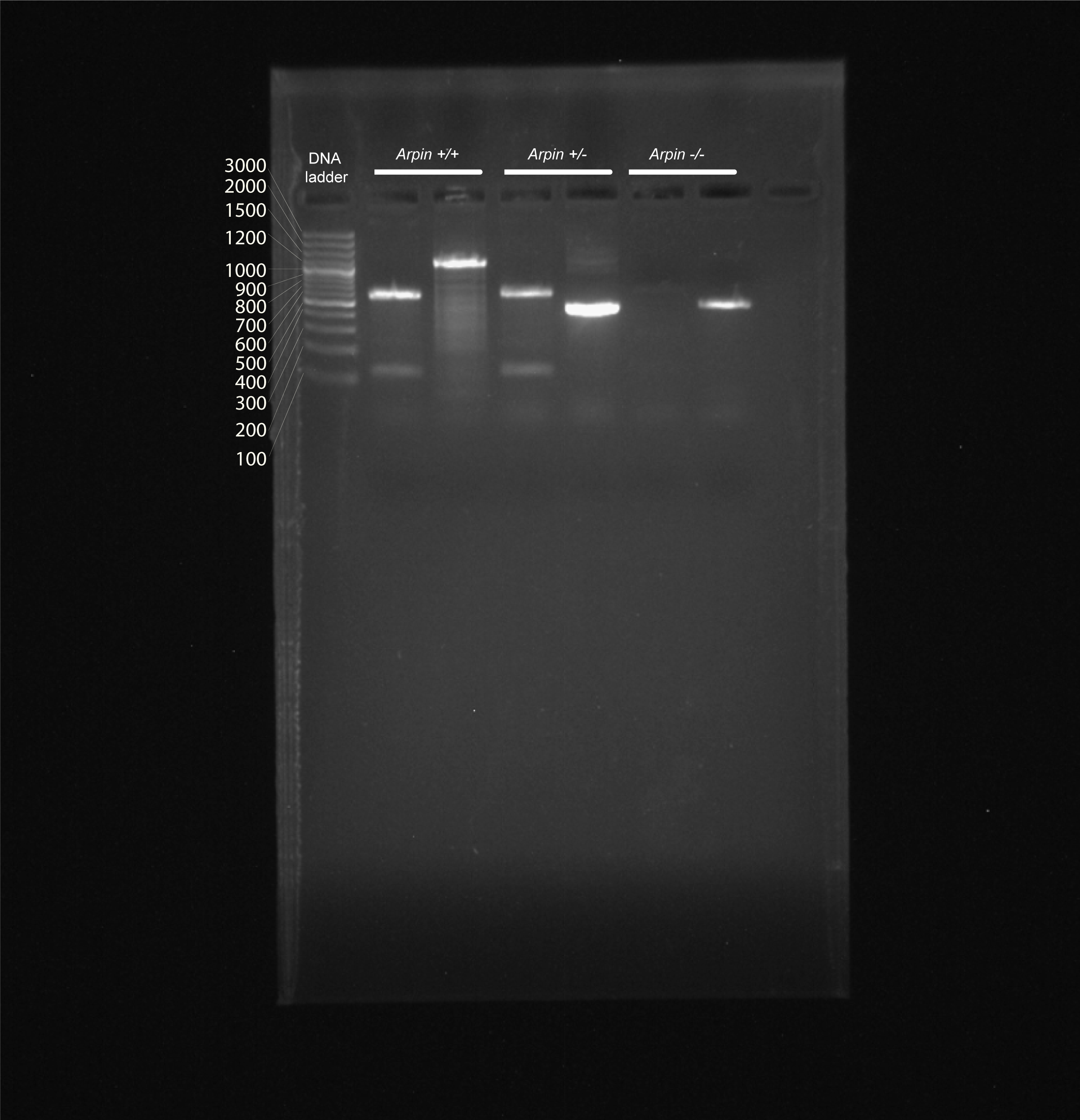

Supplement: Figure 8—source data 1. [file elife-90692-fig8-data1.zip › Figure 8 - Source data 1. Uncropped and labelled membranes for Figure 8/Fig 8B_Genotyping gel_Labelled.tif]

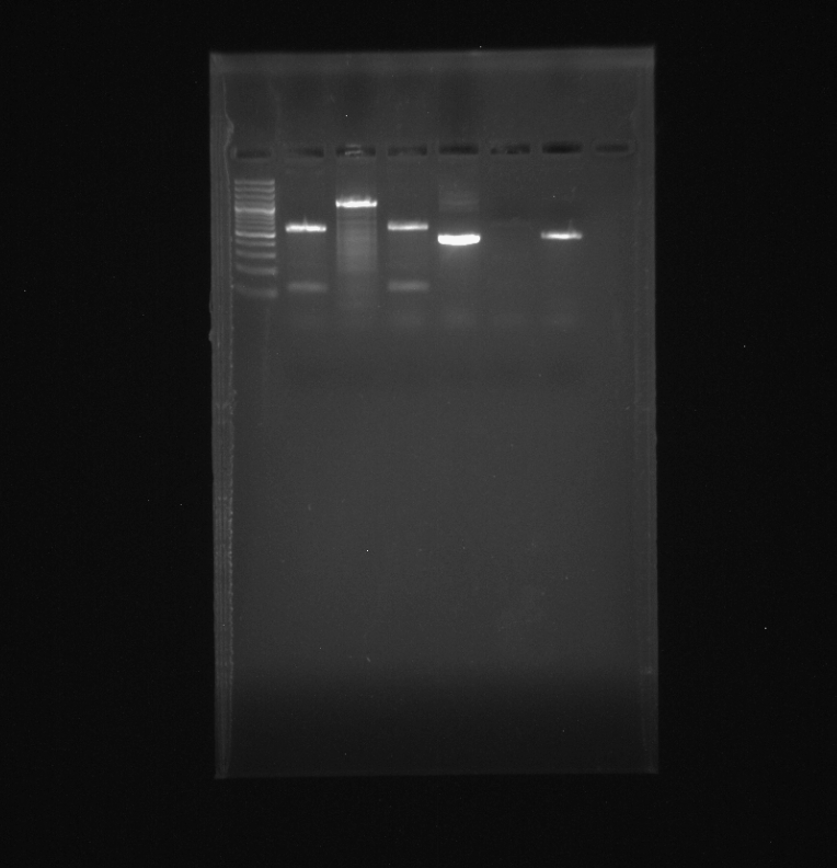

Supplement: Figure 8—source data 2. [file elife-90692-fig8-data2.zip › Figure 8 - Source data 2. Raw unedited membranes for Figure 8/Fig 8B_Genotyping gel.tif]

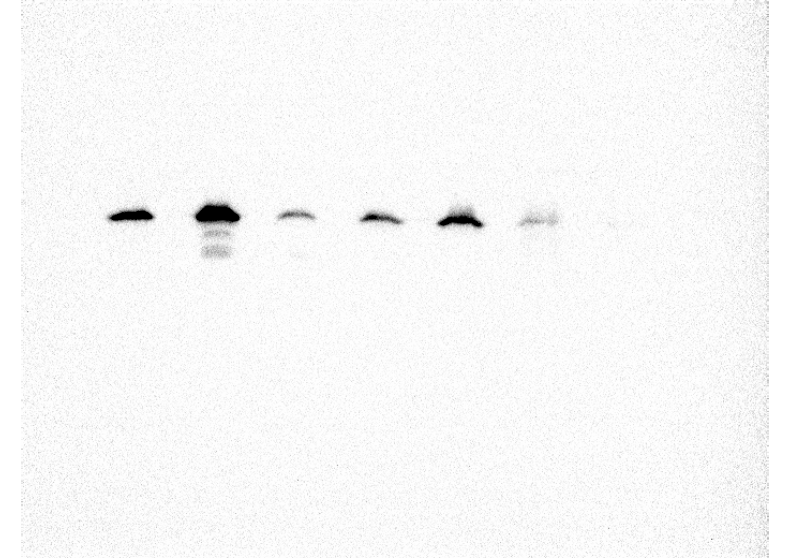

Supplement: Figure 8—source data 2. [file elife-90692-fig8-data2.zip › Figure 8 - Source data 2. Raw unedited membranes for Figure 8/Fig 8C_Arpin.tif]

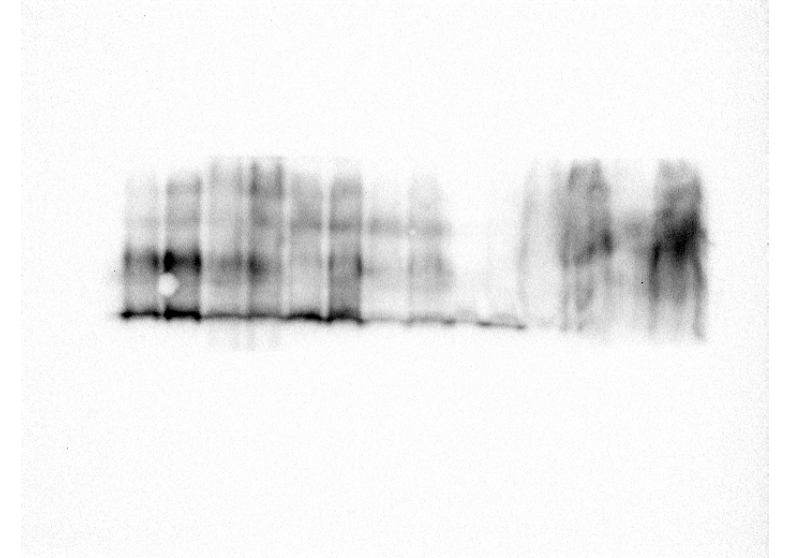

Supplement: Figure 8—source data 2. [file elife-90692-fig8-data2.zip › Figure 8 - Source data 2. Raw unedited membranes for Figure 8/Fig 8C_Gamma Tubulin.tif]

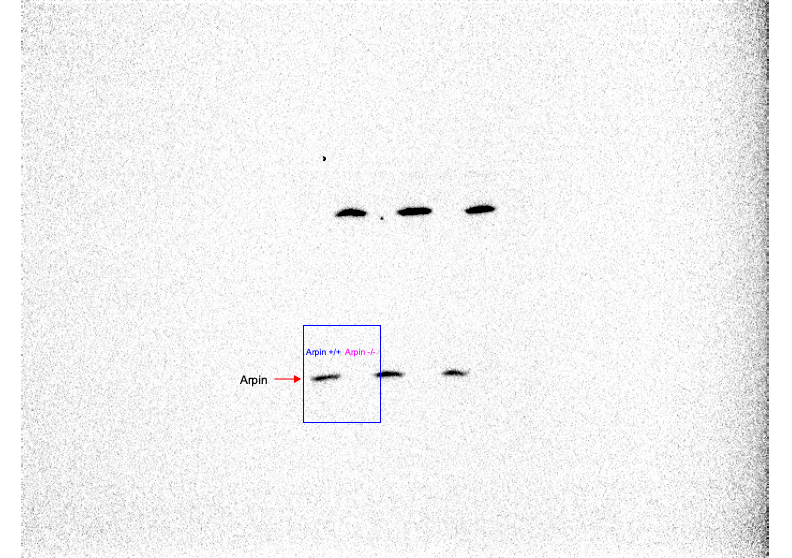

Supplement: Figure 8—figure supplement 1—source data 1. [file elife-90692-fig8-figsupp1-data1.zip › Figure 8 - Figure supplement 1 - Source data 2. Uncropped and labelled membranes for Figure 8 - Figure supplement 1/Fig 8_FS1_Arpin_Labelled.tif]

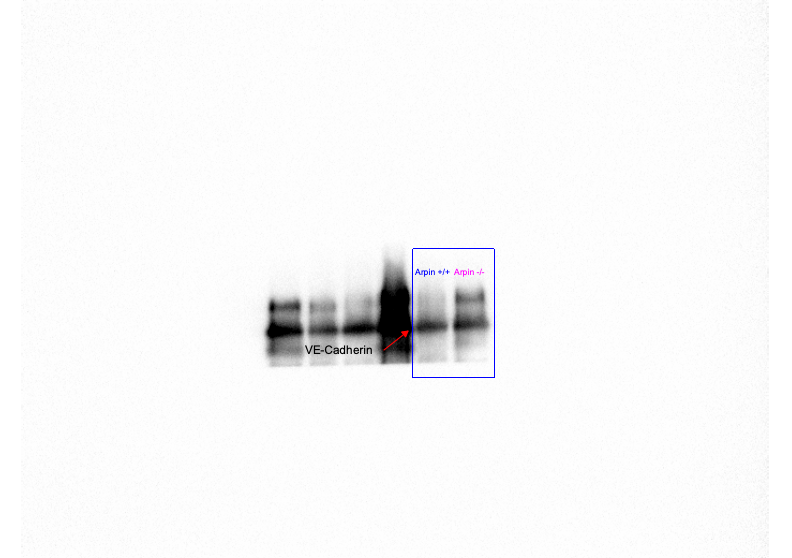

Supplement: Figure 8—figure supplement 1—source data 1. [file elife-90692-fig8-figsupp1-data1.zip › Figure 8 - Figure supplement 1 - Source data 2. Uncropped and labelled membranes for Figure 8 - Figure supplement 1/Fig 8_FS1_VE-Cadherin_Labelled.tif]

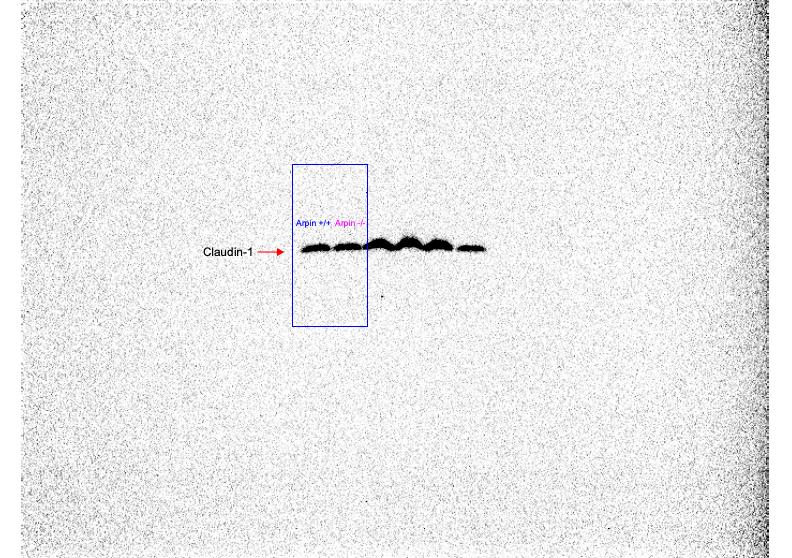

Supplement: Figure 8—figure supplement 1—source data 1. [file elife-90692-fig8-figsupp1-data1.zip › Figure 8 - Figure supplement 1 - Source data 2. Uncropped and labelled membranes for Figure 8 - Figure supplement 1/Fig 8_FS1_Claudin-1_Labelled.tif]

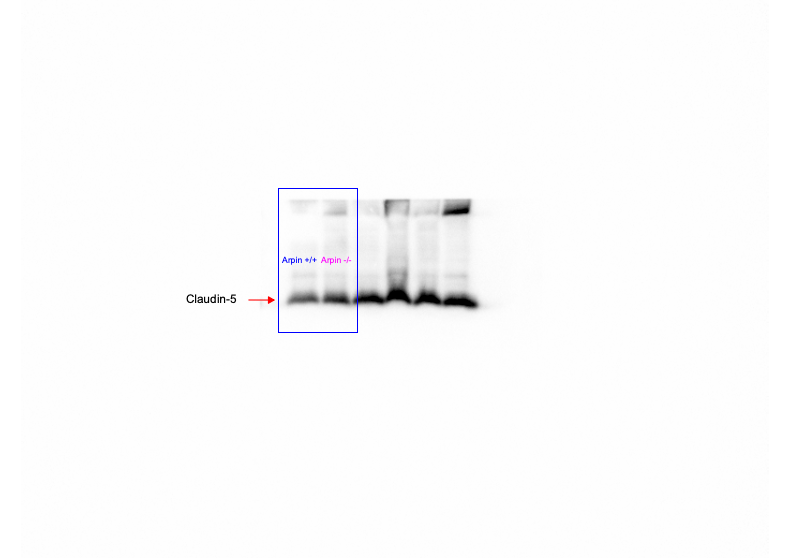

Supplement: Figure 8—figure supplement 1—source data 1. [file elife-90692-fig8-figsupp1-data1.zip › Figure 8 - Figure supplement 1 - Source data 2. Uncropped and labelled membranes for Figure 8 - Figure supplement 1/Fig 8_FS1_Claudin-5_Labelled.tif]

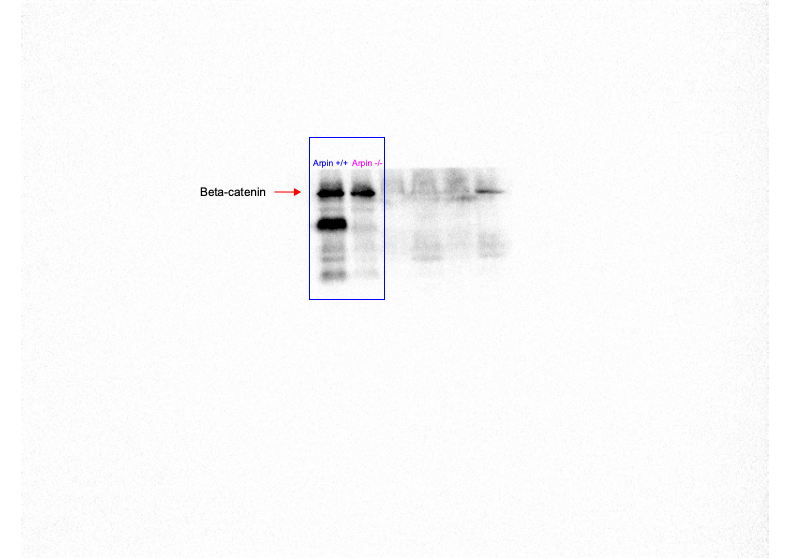

Supplement: Figure 8—figure supplement 1—source data 1. [file elife-90692-fig8-figsupp1-data1.zip › Figure 8 - Figure supplement 1 - Source data 2. Uncropped and labelled membranes for Figure 8 - Figure supplement 1/Fig 8_FS1_B-catenin_Labelled.tif]

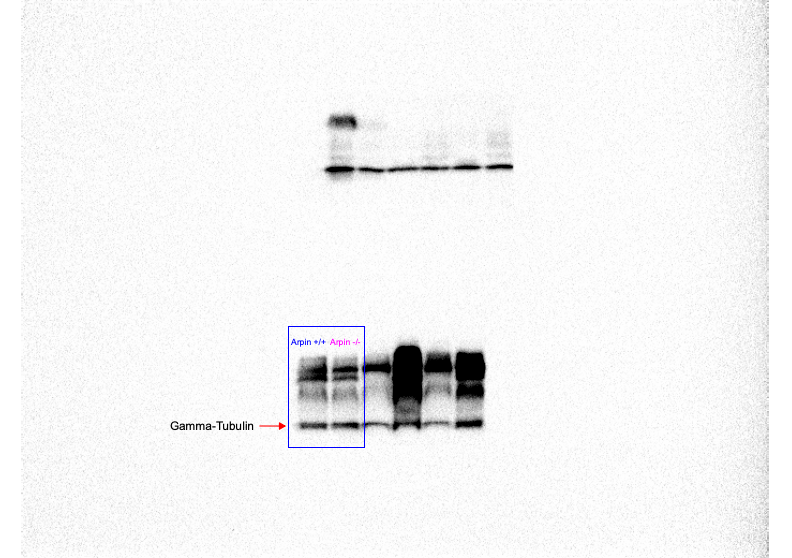

Supplement: Figure 8—figure supplement 1—source data 1. [file elife-90692-fig8-figsupp1-data1.zip › Figure 8 - Figure supplement 1 - Source data 2. Uncropped and labelled membranes for Figure 8 - Figure supplement 1/Fig 8_FS1_Gamma Tubulin_Labelled.tif]

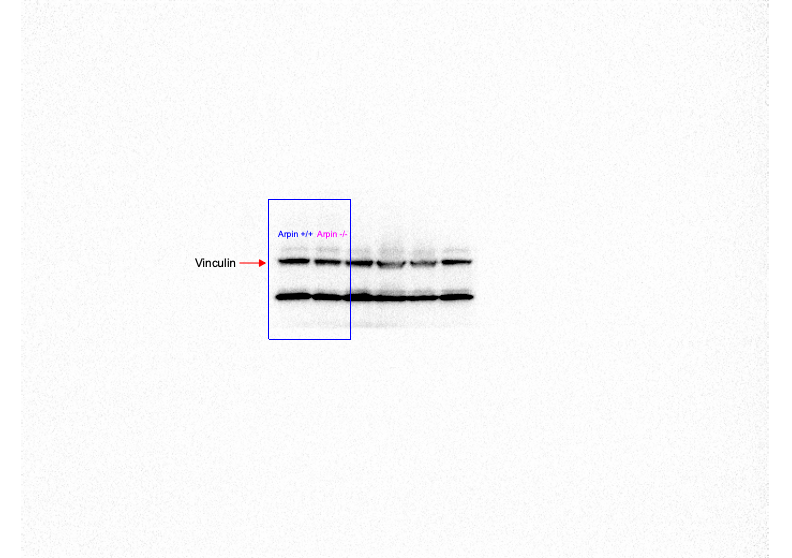

Supplement: Figure 8—figure supplement 1—source data 1. [file elife-90692-fig8-figsupp1-data1.zip › Figure 8 - Figure supplement 1 - Source data 2. Uncropped and labelled membranes for Figure 8 - Figure supplement 1/Fig 8_FS1_Vinculin_Labelled.tif]

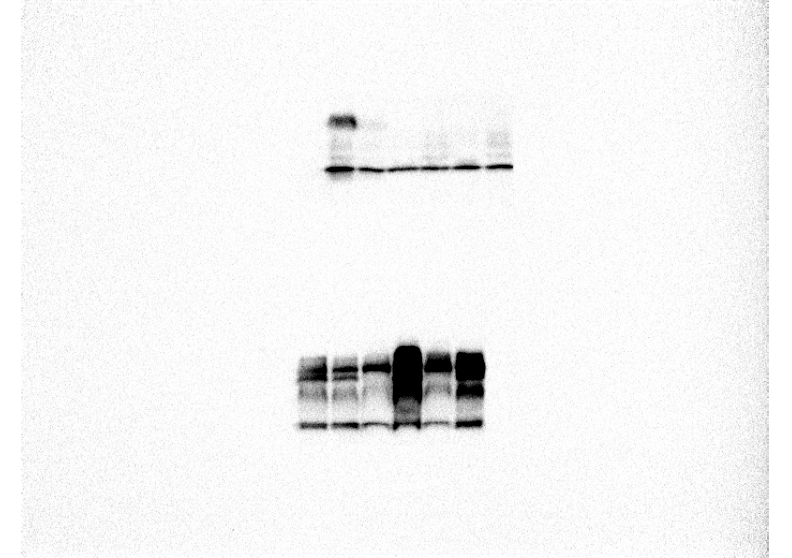

Supplement: Figure 8—figure supplement 1—source data 2. [file elife-90692-fig8-figsupp1-data2.zip › Figure 8 - Figure supplement 1 - Source data 2. Raw unedited membranes for Figure 8 - Figure supplement 1/Fig 8_FS1_Gamma Tubulin.tif]

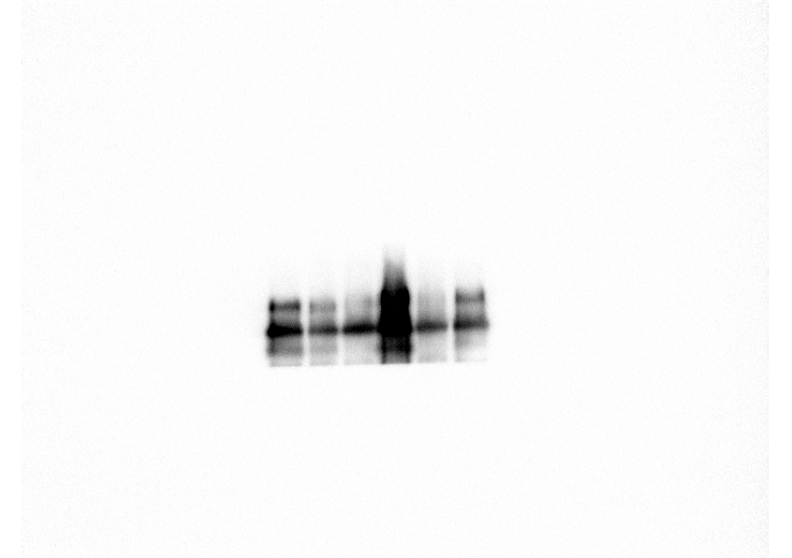

Supplement: Figure 8—figure supplement 1—source data 2. [file elife-90692-fig8-figsupp1-data2.zip › Figure 8 - Figure supplement 1 - Source data 2. Raw unedited membranes for Figure 8 - Figure supplement 1/Fig 8_FS1_VE-Cadherin.tif]

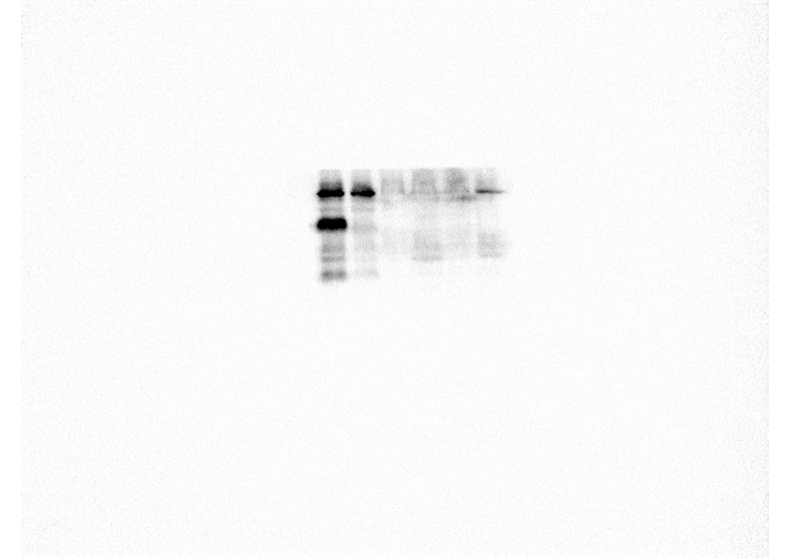

Supplement: Figure 8—figure supplement 1—source data 2. [file elife-90692-fig8-figsupp1-data2.zip › Figure 8 - Figure supplement 1 - Source data 2. Raw unedited membranes for Figure 8 - Figure supplement 1/Fig 8_FS1_B-catenin.tif]

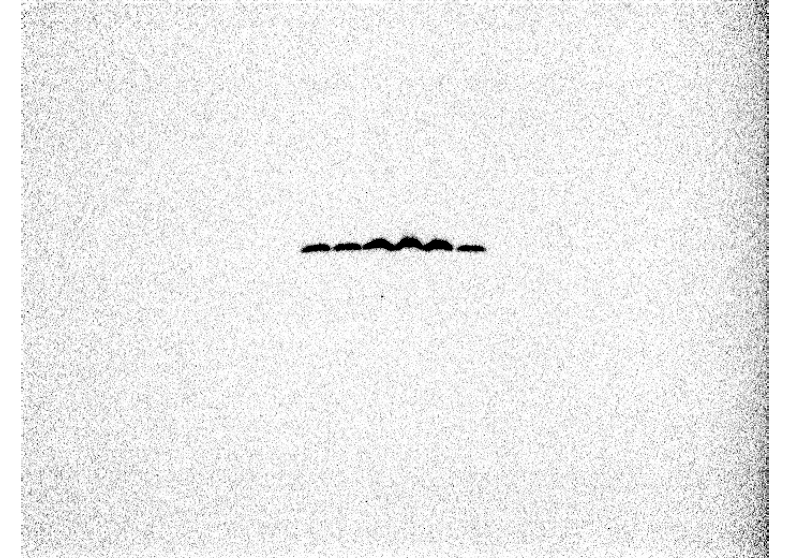

Supplement: Figure 8—figure supplement 1—source data 2. [file elife-90692-fig8-figsupp1-data2.zip › Figure 8 - Figure supplement 1 - Source data 2. Raw unedited membranes for Figure 8 - Figure supplement 1/Fig 8_FS1_Claudin-1.tif]

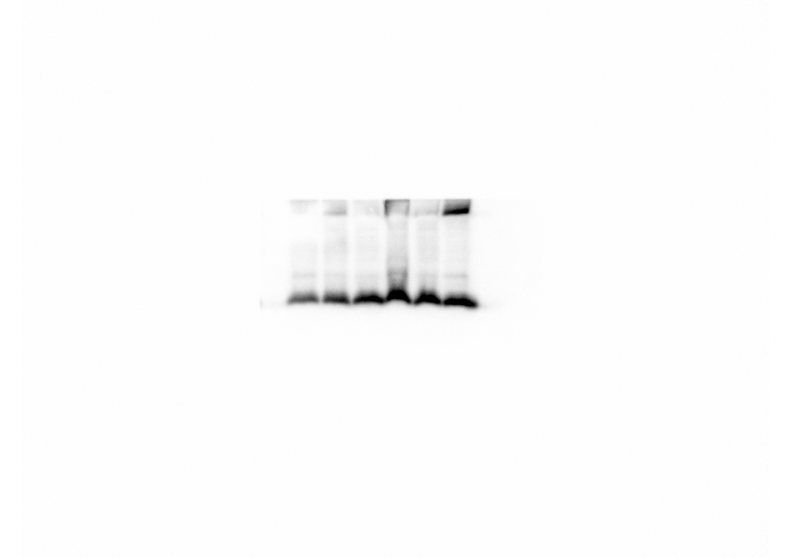

Supplement: Figure 8—figure supplement 1—source data 2. [file elife-90692-fig8-figsupp1-data2.zip › Figure 8 - Figure supplement 1 - Source data 2. Raw unedited membranes for Figure 8 - Figure supplement 1/Fig 8_FS1_Claudin-5.tif]

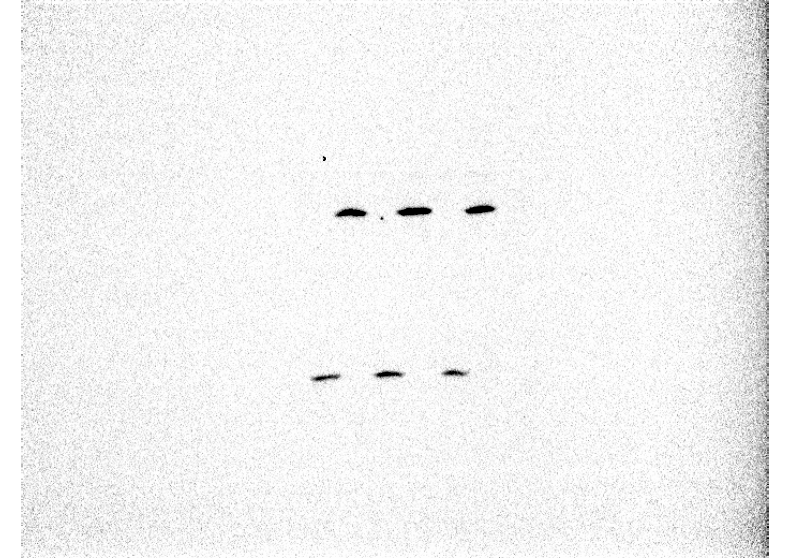

Supplement: Figure 8—figure supplement 1—source data 2. [file elife-90692-fig8-figsupp1-data2.zip › Figure 8 - Figure supplement 1 - Source data 2. Raw unedited membranes for Figure 8 - Figure supplement 1/Fig 8_FS1_Arpin.tif]

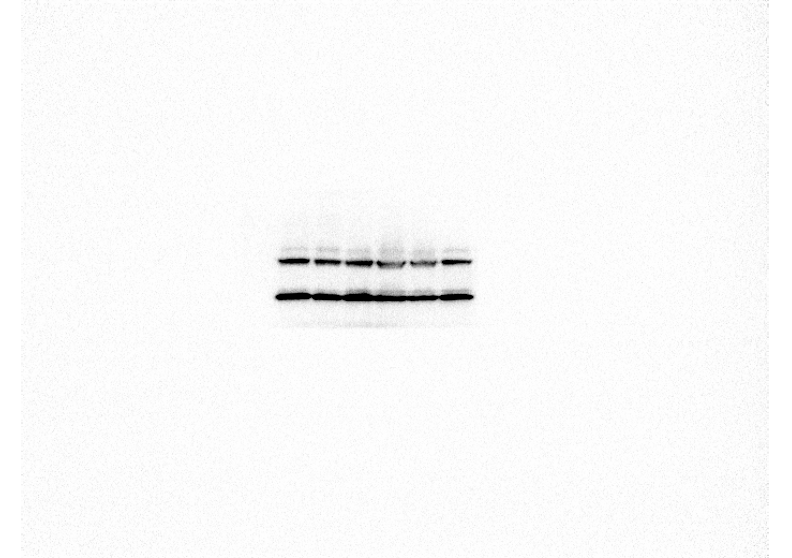

Supplement: Figure 8—figure supplement 1—source data 2. [file elife-90692-fig8-figsupp1-data2.zip › Figure 8 - Figure supplement 1 - Source data 2. Raw unedited membranes for Figure 8 - Figure supplement 1/Fig 8_FS1_Vinculin.tif]
