# Supplementary material for: Arpin deficiency increases actomyosin contractility and vascular permeability
Source: eLife. 2024 Sep 19;12:RP90692. doi: 10.7554/eLife.90692 (PMC11412691; doi:10.7554/eLife.90692)
Supplement: Supplementary file 1. — Genotyping of 126 littermates from breedings of heterozygous (Arpin+/-) mice are shown. χ2 test probability is shown. Deviations from Mendelian rules are non-significant. [file elife-90692-supp1.docx]

**Supplementary file 1a. Table 1 - Mating statistics of arpin^+/-^ mice**

|  | **Expected (Theoretical)** | **Observed (Real)** | **Probability (**$\boldsymbol{\chi}^{\mathbf{2}}$**)** | **Degrees of freedom** | **P value** |
| --- | --- | --- | --- | --- | --- |
| Male | 63 | 66 | 0.286 | 1 | 0.5930 |
| Female | 63 | 60 |  |  |  |
| arpin^+/+^ | 31.5 | 31 | 0.540 | 2 | 0.7635 |
| arpin^+/-^ | 63 | 60 |  |  |  |
| arpin^-/-^ | 31.5 | 35 |  |  |  |
