# Supplementary material for: Arpin deficiency increases actomyosin contractility and vascular permeability
Source: eLife. 2024 Sep 19;12:RP90692. doi: 10.7554/eLife.90692 (PMC11412691; doi:10.7554/eLife.90692)
Supplement: Supplementary file 2. [file elife-90692-supp2.docx]

**Supplementary file 1b. Table 2 - Hemograms of *Arpin^+/+^* and *Arpin^-/-^* mice.**

| **Parameter** | ***Arpin^+/+^* (n=8)** | | ***Arpin^-/-^* (n=11)** | | **P value** |
| --- | --- | --- | --- | --- | --- |
|  | **mean** | **SD** | **mean** | **SD** |  |
| Leukocytes (x10^9^ cells/L) | 3.163 | 1.195 | 2.555 | 1.069 | 0.2599 |
| Neutrophils (x10^9^ cells/L) | 0.787 | 0.790 | 0.527 | 0.349 | 0.3425 |
| Lymphocytes (x10^9^ cells/L) | 2.35 | 1.14 | 1.96 | 0.90 | 0.4085 |
| Monocytes (x10^9^ cells/L) | 0.025 | 0.046 | 0.045 | 0.052 | 0.3897 |
| Erythrocytes (x10^12^ cells/L) | 7.53 | 0.72 | 7.84 | 0.59 | 0.3344 |
| Platelets (x10^9^ cells/L) | 852.0 | 200.3 | 738.1 | 357.3 | 0.4520 |
| Hemoglobin (g/L) | 110.9 | 7.6 | 113.2 | 6.9 | 0.5015 |
| Hematocrit (L/L) | 0.25 | 0.03 | 0.24 | 0.021 | 0.7500 |
